# Endogenous reduction of miR‐185 accelerates cardiac function recovery in mice following myocardial infarction via targeting of cathepsin K

**DOI:** 10.1111/jcmm.14016

**Published:** 2018-11-18

**Authors:** Chuan‐Chang Li, Xue‐Ting Qiu, Quan Sun, Ji‐Peng Zhou, Hui‐Jun Yang, Wan‐Zhou Wu, Ling‐Fang He, Can‐E Tang, Guo‐Gang Zhang, Yong‐Ping Bai

**Affiliations:** ^1^ Department of Geriatric Medicine Xiangya Hospital Central South University Changsha China; ^2^ National Clinical Research Center for Geriatric Disorder Xiangya Hospital Central South University Changsha China; ^3^ Department of Cardiovascular Medicine Xiangya Hospital Central South University Changsha China; ^4^ Institute of Medical Science Research Xiangya Hospital Central South University Changsha China

**Keywords:** acute coronary syndrome, angiogenesis, cathepsin K, hypoxia, MiR‐185, myocardial infarction

## Abstract

Angiogenesis is critical for re‐establishing the blood supply to the surviving myocardium after myocardial infarction (MI) in patients with acute coronary syndrome (ACS). MicroRNAs are recognised as important epigenetic regulators of endothelial function. The aim of this study was to determine the roles of microRNAs in angiogenesis. Eighteen circulating microRNAs including miR‐185‐5p were differently expressed in plasma from patients with ACS by high‐throughput RNA sequencing. The expressional levels of miR‐185‐5p were dramatically reduced in hearts isolated from mice following MI and cultured human umbilical vein endothelial cells (HUVECs) under hypoxia, as determined by fluorescence in situ hybridisation and quantitative RT‐PCR. Evidence from computational prediction and luciferase reporter gene activity indicated that cathepsin K (CatK) mRNA is a target of miR‐185‐5p. In HUVECs, miR‐185‐5p mimics inhibited cell proliferations, migrations and tube formations under hypoxia, while miR‐185‐5p inhibitors performed the opposites. Further, the inhibitory effects of miR‐185‐5p up‐regulation on cellular functions of HUVECs were abolished by CatK gene overexpression, and adenovirus‐mediated CatK gene silencing ablated these enhancive effects in HUVECs under hypoxia. In vivo studies indicated that gain‐function of miR‐185‐5p by agomir infusion down‐regulated CatK gene expression, impaired angiogenesis and delayed the recovery of cardiac functions in mice following MI. These actions of miR‐185‐5p agonists were mirrored by in vivo knockdown of CatK in mice with MI. Endogenous reductions of miR‐185‐5p in endothelial cells induced by hypoxia increase CatK gene expression to promote angiogenesis and to accelerate the recovery of cardiac function in mice following MI.

## 1 INTRODUCTION

Acute coronary syndrome (ACS) remains a major cause of morbidity and mortality worldwide, in which ischaemic complication represents the leading cause in cardiovascular diseases, such as hypertension and atherosclerosis.^1^ Angiogenesis is critical for re‐establishing the blood supply to the surviving myocardium after myocardial infarction (MI) and, consequently, to the recovery of cardiac function.^2^ In response to pro‐angiogenic stimuli, vascular endothelial cells need to be activated rapidly to migrate to distant sites and proliferate to form new primary capillaries from existing ones.^3^, ^4^ Impaired angiogenic responses of endothelial cells have been linked to poor cardiac function and outcome after ACS clinically. Therefore, better understandings of the molecular mechanisms of angiogenesis provide a basis for therapeutic application in ACS patients.

MicroRNA is a kind of small, nonprotein‐coding RNA that binds to specific 3′ untranslated regions (3′‐UTR) of mRNA and represses target gene expression through inhibition of translation or transcript degradation.^5^ In vascular cells, several microRNAs, such as miR‐126, miR‐217, miR‐10a, miR‐124, etc., have been identified to regulate multiple cellular functions including the glucose and lipid metabolisms, senescence and blood flow.^6^, ^7^, ^8^, ^9^ We have previously reported that miR‐15b‐5p regulates collateral artery formation by targeting protein kinase B in endothelial cells and miR‐199 regulates normalise arachidonic acid metabolism in nitrate tolerance by prostaglandin synthase.^10^, ^11^ Interestingly, endogenous expressions of miR‐133a and miR‐130, are null or very low in quiescent endothelial cells, while their expressions are ectopically induced by risk factors, resulting in endothelial dysfunction.^12^, ^13^ These results indicate that expressional alterations of endogenous microRNAs are crucial to regulations of endothelial functions. However, the roles of microRNA in hypoxia‐induced angiogenesis in endothelial cells remain poorly understood.

Based on the aforementioned studies, we tested the hypothesis that hypoxia affects endogenous microRNA expressions to promote angiogenesis in ischaemic heart. In this study, we firstly got 18 differentially expressed circulating microRNAs in plasma from ACS patients by using high‐throughput sequencing and then identified the function of miR‐185‐5p as a key regulator of angiogenesis by targeting cathepsin K (CatK), which plays an important role in regulating vascular repair.^14^ In vivo studies demonstrated that endogenous reduction of miR‐185‐5p was vital to the recovery of heart function after ischaemia in mice with MI. In perspectives, targeting miR‐185‐5p or CatK should be taken into consideration when treating ACS patients.

## 2 MATERIALS AND METHODS

A full description of materials and methods used, including patient recruitments, high‐throughput RNA sequencing, real‐time reverse transcription PCR (qPCR), induction of MI in mice, agomir infusion to mice, adenovirus infection, fluorescence in situ hybridisation (FISH), echocardiography, masson's trichrome staining, immunohistochemistry (IHC), immunofluorescence (IFC), cell cultures, plasmid transfection, luciferase reporter assay, western blot, cell proliferations, cell migrations, tube formation and statistical analysis were available as Supplemental Materials.

### 2.1 Patient recruitments

Patients who underwent coronary angiography were enrolled from the cardiac catheterisation room of Xiangya Hospital, Central South University, Hunan Province, China during 2014 and 2016. Thirty patients with ACS were selected with previously typical unstable angina pectoris or diagnosed as MI. A significant stenosis was defined as diameter stenosis ≥90% through coronary angiography. The patients were excluded if they had other potentiality that could influence neovascularisation, like symptomatic peripheral arterial diseases, decompensated heart failure, any concomitant inflammation or infectious diseases, neoplastic diseases and severe liver and kidney dysfunctions. Thirty patients who had no angina pectoris and coronary angiography revealed stenosis as diameter stenosis <50% were chosen as control. We documented all the cardiac history and risk factors. Informed consent was obtained from all participants. The study protocol was approved by the Ethics Committee of Xiangya Hospital, Central South University, Changsha, Hunan, China.

### 2.2 High‐throughput RNA sequencing

As described previously,^15^, ^16^ total RNA extracted from plasma was used to prepare the miRNA sequencing library by using an Agilent 2100 Bioanalyzer. Next, cluster generation was performed on the Illumina cBot using the TruSeq Rapid SR cluster kit (#GD‐402‐4001; Illumina, San Diego, CA, USA). The DNA fragments in the libraries were denatured with 0.1 mol/L NaOH to generate single−stranded DNA molecules. They were then observed on Illumina flow cells, amplified in situ and sequenced for 36 cycles with an Illumina HiSeq 2000 (Illumina), according to the manufacturer's instructions.

### 2.3 Statistical analysis

All quantitative results are expressed as mean ± SEM. Comparisons between two groups were analysed by unpaired Student's *t* test. Multiple comparisons were analysed with a one‐way anova followed by Tukey post‐hoc tests or Bonferroni post‐hoc analyses. Categorical variables were compared by the chi‐squared test. Statistical analysis was conducted using IBM SPSS statistics 20.0 (IBM Corp., Armonk, NY, USA) and *P* < 0.05 were considered as statistical significance.

## 3 RESULTS

### 3.1 Eighteen circulating microRNAs including miR‐185‐5p are differentially expressed in plasma from ACS patients

**Figure 1:**
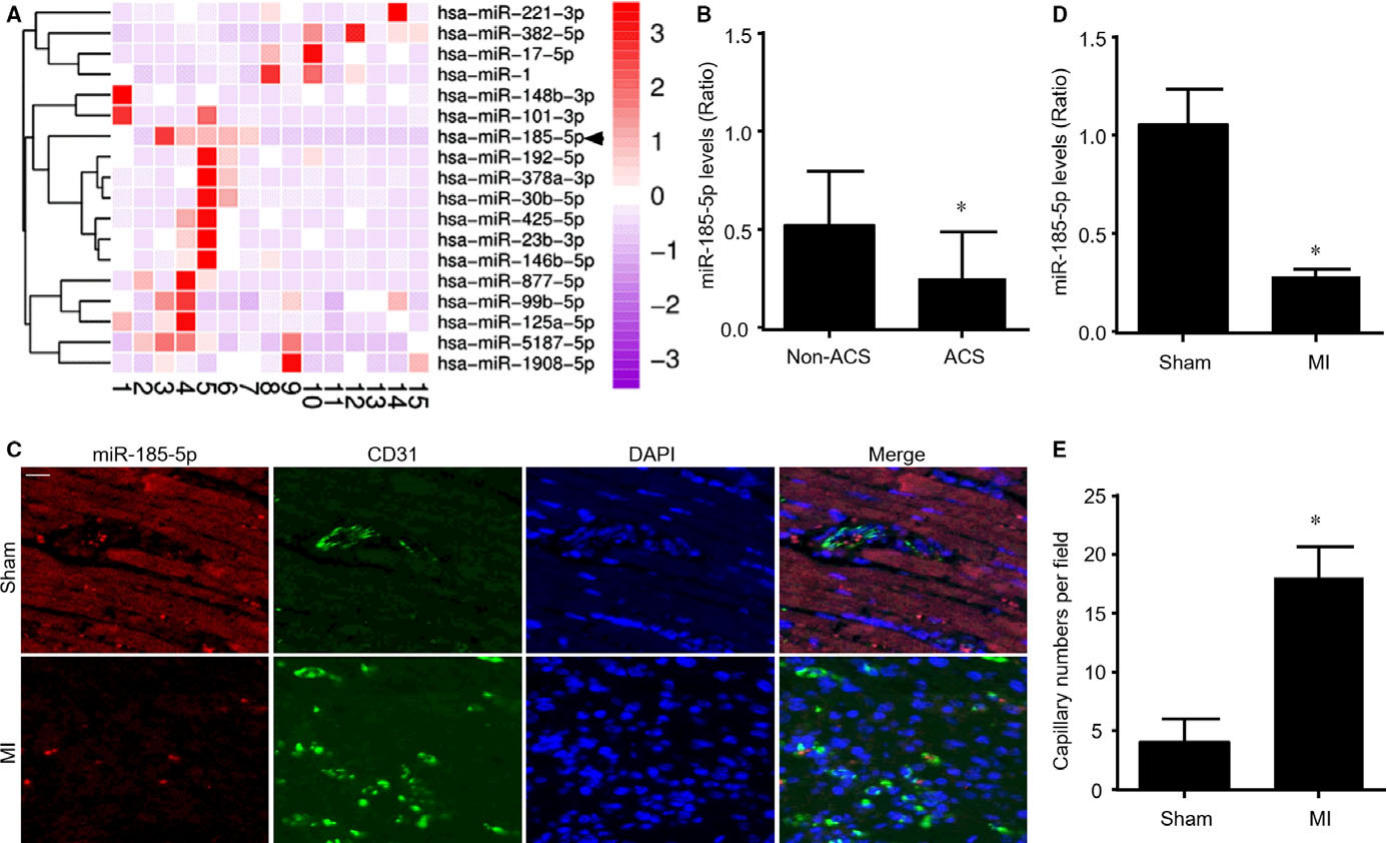
The expressional levels of miR‐185‐5p are decreased in plasma from patients with ACS and hearts isolated from mice following MI. (A) Plasma from five human individuals without ACS (number 1‐5) and 10 patients with ACS (number 6‐15) were subjected to perform high‐throughput microRNA sequencing. Eighteen differentially expressed miRNAs with 2‐fold changes or higher were shown in the heat map of hierarchical RNA clustering. (B) The plasma levels of miR‐185‐5p were detected in 30 human individuals without ACS and 30 ACS patients by real‐time RT‐PCR. The demographic data were presented in Table S1. *P < 0.05 vs non‐ACS. (C‐E) FISH analysis of miR‐185‐5p in hearts were performed in mice following MI after 7 postoperative days in C. Red, miR‐185‐5p. Green, CD31. Blue, DAPI. Scar bar is 50 μm. Quantitative analyses of miR‐185‐5p levels in D and capillary intensities in E were conducted in data from C. N = 10 per group. *P < 0.05 vs Sham

To identify which microRNAs were dysregulated in patients with ACS, we firstly collected plasma from 10 patients with ACS and five patients without ACS. High‐throughput RNA sequencing was performed to detect the expressional profiles of microRNAs in plasma and we got 18 differentially expressed microRNAs (Figure 1A). Among these altered microRNAs, six microRNAs (hsa‐miR‐221‐3p, hsa‐miR‐382‐5p, hsa‐miR‐17‐5p, hsa‐miR‐1, hsa‐miR‐5187‐5p and hsa‐miR‐1908‐5p) were increased, while 12 microRNAs (hsa‐miR‐148b‐3p, hsa‐miR‐101‐3p, hsa‐miR‐185‐5p, hsa‐miR‐192‐5p, hsa‐miR‐378a‐3p, hsa‐miR‐30b‐5p, hsa‐miR‐425‐5p, hsa‐miR‐23b‐3p, hsa‐miR‐146b‐5p, hsa‐miR‐877‐5p, hsa‐miR‐99b‐5p, hsa‐miR‐125a‐5p, hsa‐miR‐5157‐5p) were decreased.

Because miR‐185‐5p has been reported to inhibit angiogenesis and response to hypoxia,^17^, ^18^ we chose miR‐185‐5p as a candidate to regulate angiogenesis and the recovery of heart function after MI. Therefore, we confirmed the plasma levels of miR‐185‐5p by qPCR in 30 ACS patients (the demographic data were presented in Table S1). As indicated in Figure 1B, plasma miR‐185‐5p levels were down‐regulated in patients with ACS, compared to patients without ACS.

### 3.2 MiR‐185‐5p is reduced in ischaemic heart in mice following MI

To determine whether miR‐185‐5p is vital to ischaemia‐induced angiogenesis in heart, we performed FISH analysis to measure miR‐185‐5p expression in mice heart at 7 post‐MI days. As depicted in Figure 1C, miR‐185‐5p expression was positively expressed in both cardio myocytes and endothelial cells under physiological condition. While, miR‐185‐5p expression in heart was remarkably reduced in ischaemic heart in mice with MI. The reduction of miR‐185‐5p expression in ischaemic heart was further confirmed by qPCR analysis (Figure 1D). Further, decreased miR‐185‐5p expression was associated with increased capillary intensity (Figure 1E), as indicated by quantitative analysis of CD31. These data suggest that miR‐185‐5p reduction may contribute to ischaemia‐induced angiogenesis in heart.

### 3.3 Hypoxia reduces miR‐185‐5p expression in endothelial cells

**Figure 2:**
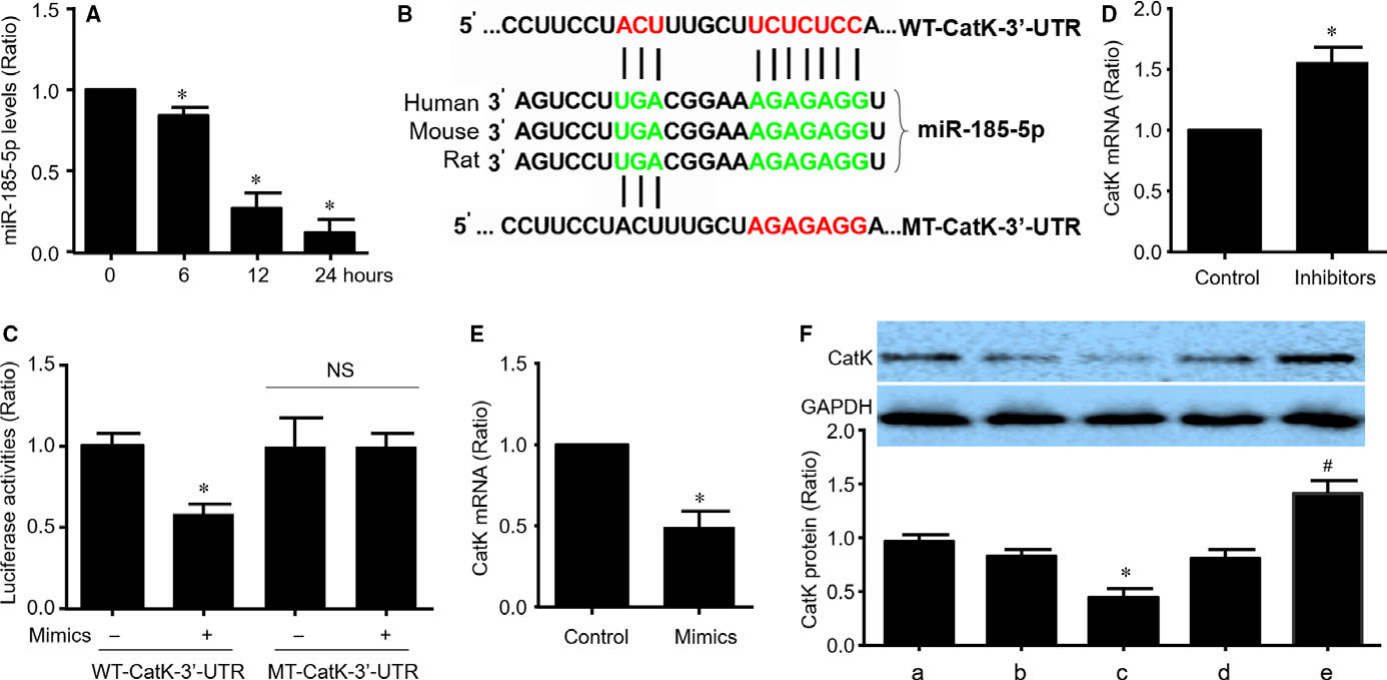
Cathepsin K (CatK) mRNA is a target of miR‐185‐5p in endothelial cells. (A) Cultured HUVECs were exposed to hypoxic conditions as indicated time and miR‐185‐5p expressional levels were assayed by real‐time RT‐PCR. N = 5 per group. *P < 0.05 vs control (point 0). (B) The highly conserved putative target site of miR‐185‐5p in 3′‐UTR of CatK mRNA was predicted by target scan analysis. (C) Plasmids of luciferase reporter gene construction containing the full‐length wild‐type 3′‐UTR of CatK mRNA (WT‐CatK‐3′‐UTR) and mutated 3′‐UTR of CatK mRNA (MT‐CatK‐3′‐UTR) were generated and transfected into HEK293 cells with control or miR‐185‐5p mimics. The luciferase activity in total cell lysates was assayed. N = 5 per group. *P < 0.05 vs WT‐CatK‐3′‐UTR alone. NS indicates no significance. (D and E) HUVECs were transfected with miR‐185‐5p inhibitors in D or mimics in E for 48 h. Cells were subjected to detect the levels of CatK mRNA by real‐time RT‐PCR. N = 5 per group. *P < 0.05 vs control. (F) HUVECs were transfected with miR‐185‐5p mimics and inhibitors for 48 h. Total cell lysates were subjected to determine the protein levels of CatK by Western blot. a, Control. b, Control mimics. c, miR‐185‐5p mimics. d, Control inhibitors. e, miR‐185‐5p inhibitors. N = 5 per group. *P < 0.05 vs control mimics (b). ^#^ *P* < 0.05 vs control inhibitors (d)

As the key cells of endothelial cells in angiogenesis,^19^ we next examined the effects of ischaemia on miR‐185‐5p expressions in cultured human umbilical vein endothelial cells (HUVECs). Ischaemia was simulated by hypoxia, as confirmed by positive expression of hypoxia inducible factor 1 α in HUVECs (Figure S1). In Figure 2A, hypoxia noticeably reduced the levels of miR‐185‐5p in a time‐dependent manner, suggesting that endogenous expression of miR‐185‐5p is suppressed in endothelial cells under hypoxia.

### 3.4 CatK is a target of miR‐185‐5p in endothelial cells

In order to investigate how miR‐185‐5p regulates angiogenesis under hypoxia, we used TargetScan database to predict the potential target of miR‐185‐5p. Computational target scan analysis showed that a highly conserved putative target site of miR‐185‐5p located in 3′‐UTR of CatK mRNA among human, rat and mouse (Figure 2B). To examine whether miR‐185‐5p is able to repress CatK gene expression through direct 3′‐UTR interaction, we cloned wild‐type CatK 3′‐UTR (WT‐CatK‐3′‐UTR) or mutated CatK 3′‐UTR (MT‐CatK‐3′‐UTR) to luciferase reporter plasmid and performed reporter analysis in HEK293 cells. As shown in Figure 2C, the co‐transfection of miR‐185‐5p mimics with plasmid of WT‐CatK‐3′‐UTR reporter gene reduced luciferase activity. However, co‐transfection of miR‐185‐5p mimics with plasmid of MT‐CatK‐3′‐UTR reporter gene did not result in a significant inhibition of luciferase activity. These data indicated that CatK is a target of miR‐185‐5p.

To further verify if CatK is a target gene of miR‐185‐5p in endothelial cells, HUVECs were transfected with miR‐185 inhibitors or miR‐185 mimics. Inhibition of miR‐185‐5p up‐regulated CatK mRNA level (Figure 2D), while miR‐185 mimics down‐regulated CatK mRNA level (Figure 2E). Further, Western blot analysis revealed that suppression of miR‐185‐5p increased CatK protein level, while miR‐185 overexpression did opposite (Figure 2F). These data demonstrate that CatK is a target of miR‐185‐5p in endothelial cells.

### 3.5 MiR‐185‐5p inhibits cell proliferation and migration in HUVECs under hypoxia

**Figure 3:**
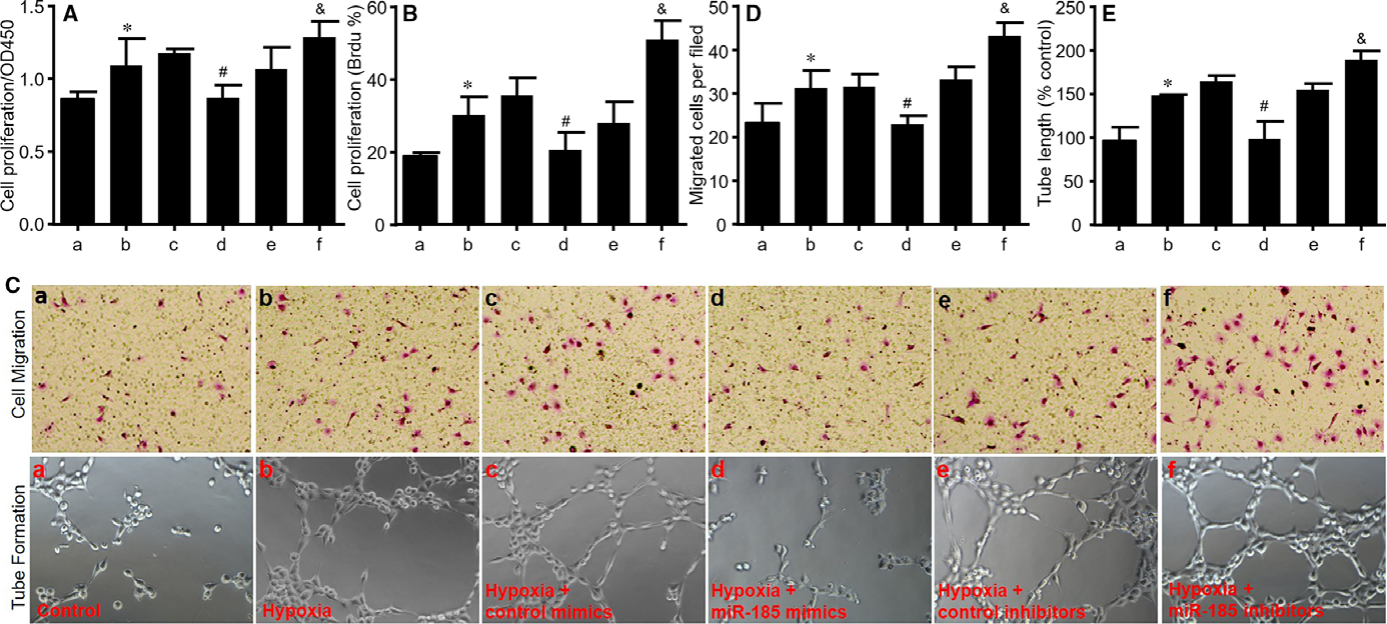
MiR‐185‐5p negatively regulates HUVECs proliferations and migrations under hypoxia. Cultured HUVECs were transfected with control mimics, miR‐185‐5p mimics (50 nmol/L), control inhibitors and miR‐185‐5p inhibitors (100 nmol/L) for 48 h followed by 24‐h hypoxia. a, Control. b, Hypoxia. c, Hypoxia plus control mimics. d, Hypoxia plus miR‐185‐5p mimics. e, Hypoxia plus control inhibitors. f, Hypoxia plus miR‐185‐5p inhibitors. (A and B) Cell proliferations were determined by CCK8 in A and BrdU in B. (C‐E) Cell migration and tube formation were assayed in HUVECs after treatments and representative pictures were shown in C. Quantitative analysis of cell migration in D and tube formation in E were performed in data from C. N = 5 per group. *P < 0.05 vs control (a). ^#^ *P* < 0.05 vs hypoxia plus control mimics (c). ^&^ *P* < 0.05 vs hypoxia plus control inhibitors (e)

Both proliferation and migration of endothelial cells are essential to the postischaemic angiogenesis.^4^ Thus, we examined whether miR‐185‐5p reduction contributes to hypoxia‐induced cell proliferation and migration in cultured HUVECs, as determined by CCK‐8 and BrdU assay (Figure S2). As presented in Figure 3A, B, gain‐function of miR‐185‐5p by specific mimics decreased cell proliferation; however, loss‐function of miR‐185‐5p by specific inhibitors further enhanced cell proliferations in HUVECs under hypoxia. Similarly, miR‐185‐5p mimics inhibited cell migrations, but miR‐185‐5p inhibitors increased cell migrations in HUVECs under hypoxia (Figure 3C, D). These findings prove that miR‐185‐5p is crucial to cell proliferation and migration during hypoxia.

### 3.6 MiR‐185‐5p impairs tube formations in HUVECs under hypoxia

Tube formation is a vital step in endothelial cell‐mediated angiogenesis.^20^ Therefore, we examined if miR‐185‐5p regulates hypoxia‐induced tube formation in HUVECs. As indicated in Figure 3C, E, miR‐185‐5p up‐regulation by mimics inhibited tube formations, but miR‐185‐5p suppression by inhibitors increased tube formations in HUVECs under hypoxia.

### 3.7 MiR‐185‐5p delays wound healing in HUVECs under hypoxia

**Figure 4:**
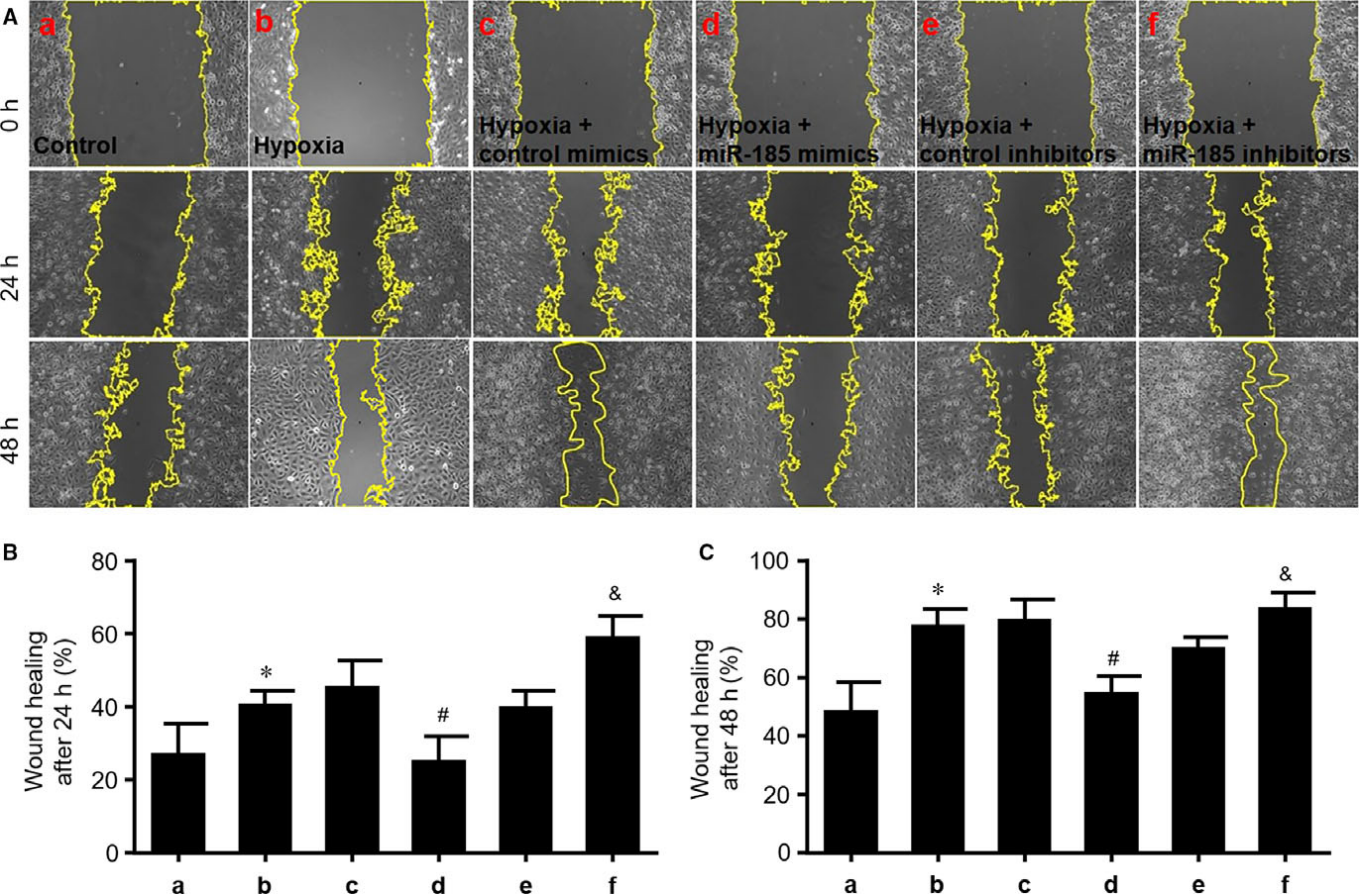
MiR‐185‐5p suppresses wound healing in cultured HUVECs. (A) Cultured HUVECs were transfected with control mimics, miR‐185‐5p mimics (50 nmol/L), control inhibitors and miR‐185‐5p inhibitors (100 nmol/L) for 48 h followed by 24‐h hypoxia. a, Control. b, Hypoxia. c, Hypoxia plus control mimics. d, Hypoxia plus miR‐185‐5p mimics. e, Hypoxia plus control inhibitors. f, Hypoxia plus miR‐185‐5p inhibitors. Wound healing was assessed in HUVECs at 24 and 48 h after scratching. (B and C) Quantitative analysis of wound healing after 24 h in B or after 24 h in C was conducted in data from A. N = 5 per group. *P < 0.05 vs control (a). ^#^ *P* < 0.05 vs hypoxia plus control mimics (c). ^&^ *P* < 0.05 vs hypoxia plus control inhibitors (e)

We next detected whether miR‐185‐5p regulates angiogenesis by performing wound healing test. The wound healing was determined at 24 and 48 hours after scratch‐induced injury in cultured HUVECs. As demonstrated in Figure 4A‐C, up‐regulation of miR‐185‐5p by mimics delayed wound healing, while miR‐185‐5p inhibitors promoted wound healing in HUVECs under hypoxia. Collectively, these data suggest that miR‐185‐5p regulates angiogenesis including cell proliferation, migration, tube formation and would healing in endothelial cells under hypoxia.

### 3.8 Overexpression of CatK abolishes the effects of miR‐185‐5p on proliferation, migration and tube formation of HUVECs

**Figure 5:**
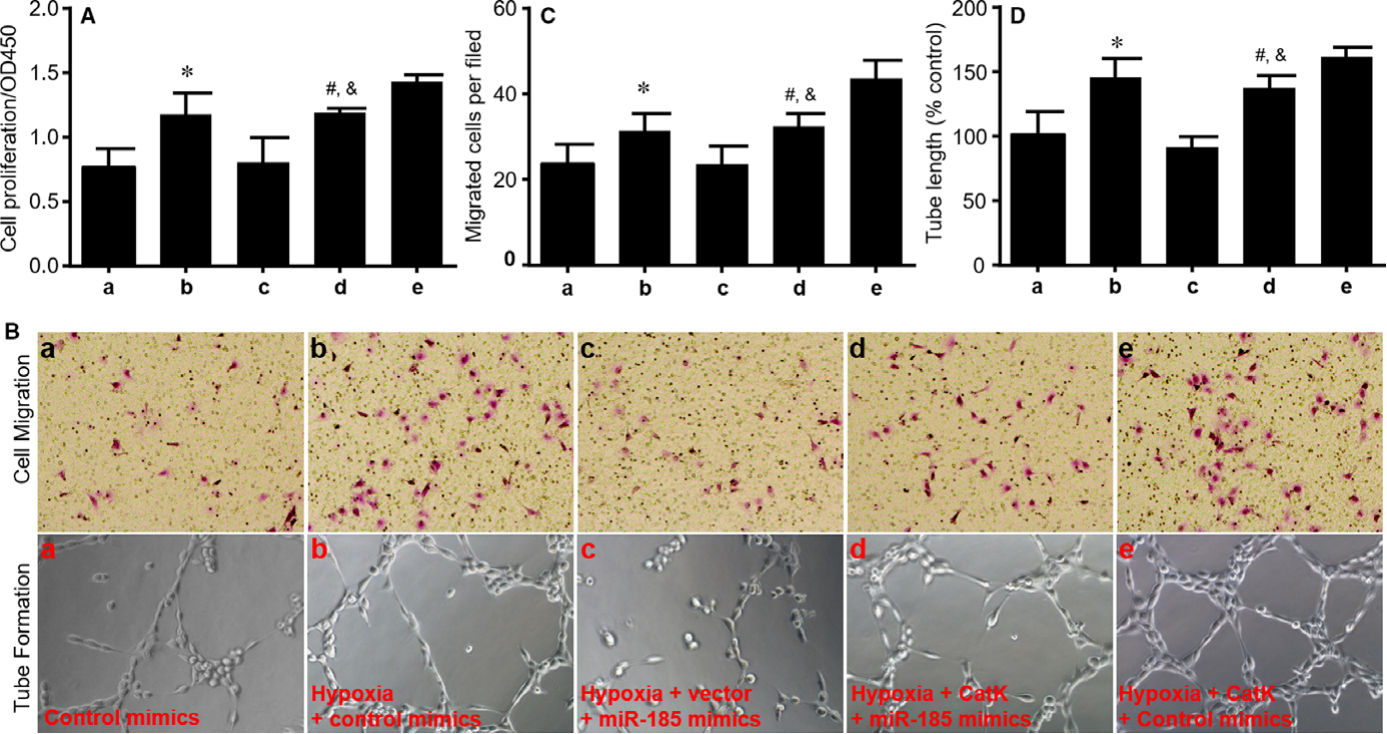
Overexpression of cathepsin K (CatK) abolishes the inhibitory effects of miR‐185‐5p on proliferation, migration and tube formation of HUVECs under hypoxia. Cultured HUVECs were infected with adenovirus expressing CatK for 48 h followed by transfection with controls and miR‐185‐5p mimics (50 nmol/L) for 24 h. Then cells were exposed to hypoxia for 24 h. a, Control mimics. b, Hypoxia plus control mimics. c, Hypoxia plus miR‐185‐5p mimics plus vector. d, Hypoxia plus miR‐185‐5p mimics plus CatK. e, Hypoxia plus control mimics plus CatK. (A) Cell proliferations were determined by CCK8. (B) Cell migration and tube formation were assayed in HUVECs after treatments and representative pictures were shown. (C and D) Quantitative analysis of cell migration in C and tube formation in D were performed in data from B. N = 5 per group. *P < 0.05 vs control mimics (a). ^#^ *P* < 0.05 vs hypoxia plus miR‐185‐5p mimics plus vector (c). ^&^ *P* < 0.05 vs hypoxia plus control mimics plus CatK (e)

To determine whether CatK mediates the effects of miR‐185‐5p on cellular functions, HUVECs infected with adenovirus expressing CatK were transfected with miR‐185‐5p mimic for 24‐48 hours followed by hypoxia. As represented, miR‐185‐5p mimic inhibited cell proliferation (Figure 5A), migration (Figure 5B, C) and tube formation (Figure 5B, D) in cultured HUVECs under hypoxia when cells were infected with adenovirus vector, but it did not produce these inhibitory effects in cells infected with adenovirus expressing CatK mRNA.

### 3.9 Silencing CatK ablates the effects of miR‐185‐5p inhibition on cell proliferations, migrations and tube formations in HUVECs

Conversely, we detected the effects of miR‐185‐5p inhibitors on cellular functions if CatK is null by infecting HUVECs with adenovirus expressing CatK shRNA. As expected, miR‐185‐5p inhibitor mirrored or enhanced hypoxia‐induced cell proliferations (Figure S3A), migrations (Figure S3B, C) and tube formations (Figure S3B, D) in cultured HUVECs. Importantly, these effects of miR‐185‐5p inhibitors were abolished by CatK shRNA but not scramble shRNA. These data support this notion that CatK acts as the downstream target of miR‐185‐5p in regulating angiogenesis.

### 3.10 CatK regulates c‐notch1, Hes1, Hey1 and Hey2 mRNA expression in HUVECs

To uncover how CatK regulates angiogenesis, we measured notch signalling, which has been reported to play critical roles in vascular development.^21^, ^22^ As shown in Figure S4A, CatK gene silencing by shRNA reduced c‐notch1 protein level, while CatK overexpression dramatically up‐regulated c‐notch1 protein level in HUVECs under hypoxia. Further, mRNA expressions of Hes1, Hey1 and Hey2, which are the downstream genes of notch,^23^, ^24^ were significantly repressed by CatK shRNA (Figure S4B), but activated by CatK gene overexpression (Figure S4C).

### 3.11 Gain‐function of miR‐185‐5p inhibits ischaemia‐induced angiogenesis in mice following MI

**Figure 6:**
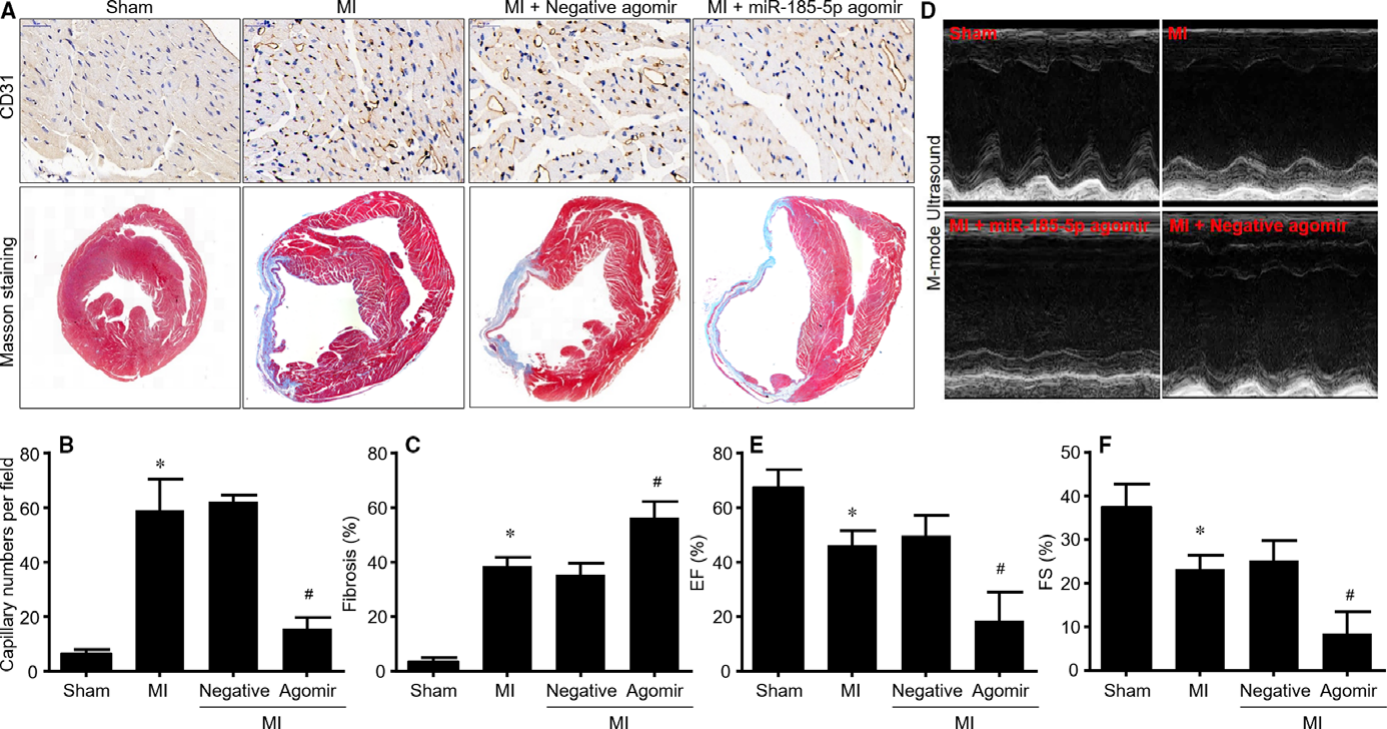
Gain‐function of miR‐185‐5p impairs angiogenesis and delays the recovery of cardiac functions in mice following MI. The animal protocols of miR‐185‐5p agomir infusion and MI model induction were shown in Figure S5A. (A‐C) IHC analysis of CD31 and masson's trichrome staining of fibrosis were performed in cross‐sections of left ventricle in mice after 28 postoperative days. Representative pictures were presented in A. Quantitation of capillary intensities in B or fibrosis in C was conducted. (D‐F) Heart functions were determined by echocardiography in mice after 28 postoperative days before scarified. Representative images of M‐mode echocardiography were shown in D. Both EF in E and FS in F were calculated. N = 10‐15 per group. *P < 0.05 vs Sham. ^#^ *P* < 0.05 vs MI plus negative agomir

Knowing that miR‐185‐5p negatively regulates proliferations, migrations and tube formations in endothelial cells by targeting CatK, we next determined whether up‐regulation of miR‐185‐5p inhibits angiogenesis in ischaemic heart in vivo. To this point, mice were infused with miR‐185‐5p agomir and were conducted with MI surgery (Figure S5A). Infusion of miR‐185‐5p agomir obviously increased miR‐185‐5p levels in hearts, compared with control agomir (Figure S5B). As indicated in Figure 6A, B, the capillary intensities in ischaemic area of hearts isolated from mice with MI after 28 postoperative days were significantly higher than hearts from mice with sham surgery, as determined by IHC analysis of CD31. Treatment of miR‐185‐5p agomir but not control agomir completely reduced capillary numbers in mice with MI. Furthermore, administration of miR‐185‐5p agomir reduced fibrosis in ischaemic heart (Figure 6A, C). These results suggest that miR‐185‐5p reduction contributes to ischaemia‐induced angiogenesis in mice after MI.

### 3.12 MiR‐185‐5p agomir delays the recovery of heart functions in mice following MI

Angiogenesis is a key regenerative event to recover the blood supply and to repair cardiac function in ACS patients.^25^ Next, we determined whether miR‐185‐5p agomir delays the recovery of heart functions in mice following MI by echocardiographic analysis (Figure 6D). Echocardiographic parameters were shown in Table S2. Both EF and FS were dramatically decreased in mice with MI, which were further reduced by miR‐185‐5p agomir, compared to mice with negative agomir treatment (Figure 6E, F).

### 3.13 Gain‐function of miR‐185‐5p reduces CatK protein expression in mice following MI

We next examined whether miR‐185‐5p represses CatK gene expression in vivo. As shown in Figure S6A, B, IFC analysis indicated that the numbers of both CatK^+^ and CD31^+^ cells in ischaemic area were significantly enhanced in heart isolated from mice with MI, compared to mice with sham. Administration of miR‐185‐5p agomir but not negative agomir extensively repressed CatK protein expressions in CD31^+^ cells in mice after 28 postoperative days. These effects were further confirmed by qPCR analysis showing the mRNA expression of CatK in ischaemic area was significantly reduced by miR‐185‐5p agomir in mice with MI surgery (Figure S6C).

### 3.14 Knockdown of CatK impairs angiogenesis and delays the recovery of heart functions in mice following MI

Above, we have provided evidence to reveal that CatK mRNA is a target of miR‐185‐5p. As a result, we thought that silencing of CatK would mirror the effects of miR‐185‐5p agomir in mice with MI. To test this notion, in vivo loss‐function of CatK was induced by infecting mice with adenovirus expressing CatK shRNA (Figure S5C, D). Similar to miR‐185‐5p agomir, CatK gene silencing decreased capillary intensities and increased fibrosis in ischaemic area of hearts isolated from mice with MI after 28 postoperative days, compared to mice infected with adenovirus expressing scramble shRNA (Figure S7A‐C, G, H).

We also detected the effects of CatK gene silencing on cardiac function in mice with MI by echocardiography and the echocardiographic parameters were shown in Table S3. As expected, knockdown of CatK gene expression reduced EF and FS in mice infected with adenovirus expressing CatK shRNA at 28 postoperative days after MI surgery (Figure S7D‐F). Taking these data together, it reveals that miR‐185‐5p/CatK axis plays a key role in angiogenesis and repair of heart function after MI.

### 3.15 MiR‐185‐5p agonist and CatK inhibition suppress arteriogenesis in ischaemic heart isolated from mice with MI

Growth of functional arteries from angiogenesis, which is driven by flow shear stress, is essential for the restoration of blood flow to ischaemic organs.^26^, ^27^ Finally, we examined the roles of miR‐185‐5p and CatK in arteriogenesis in mice heart by performing double staining of CD31 and ɑ‐SMA shown in Figure S8A, B, either miR‐185‐5p agonist or CatK shRNA noticeably decreased arterial numbers in ischaemic hearts from mice after 28 postoperative days.

## 4 DISCUSSION

In this study, we have found that 18 circulating microRNAs including miR‐185‐5p were differently expressed in patients with ACS. We also demonstrated that endogenous reduction of miR‐185‐5p expression in endothelial cells during hypoxia up‐regulated CatK gene expression and activated multiple cellular functions such as proliferation, migration and tube formation. In vivo gene manipulations of miR‐185‐5p up‐regulation by agomir or CatK down‐regulation by adenovirus‐mediated RNA interference impaired angiogenesis and delayed the recovery of cardiac function in mice with MI. To the best of our knowledge, this study is firstly to report that the miR‐185‐5p/CatK axis plays a key role in revascularisation after MI.

The major discovery of the present study is that CatK is a target of miR‐185‐5p in endothelial cells. MiR‐185, located in the 22q11.2 gene locus, is generally regarded as a regulator involved in the biological processes of carcinoma cells and neurological disorders by targeting marginal zone B and B1 cell‐specific protein, etc.^28^ Several studies have explored the role of CatK in different models of heart failure induced by high‐fat diet, pressure overload, ageing and diabetes.^29^, ^30^, ^31^, ^32^ However, the potential mechanisms by which CatK regulates the cardiac function after MI remains unknown. Here, we exploited that CatK is a novel target of miR‐185‐5p in endothelial cells. This was demonstrated by the following evidence: (a) miR‐185‐5p target site, which belongs to conserved groups, located in the 3′‐UTR of CatK mRNA as indicated “ACUXXXXXUCUCUCC”; (b) Transfection of miR‐185‐5p decreased luciferase reporter activity in plasmid of WT‐CatK‐3′‐UTR, but not in plasmid of CatK‐3′‐UTR with the mutant of miR‐185‐5p target site; (c) Overexpression of miR‐185‐5p decreased CatK mRNA level, while inhibition of miR‐185‐5p increased CatK gene expression in HUVECs; (d) Importantly, the actions of miR‐185‐5p agomir were compromised by CatK gene overexpression in endothelial cells and were mirrored by CatK gene silencing in mice with MI.

Besides regulating angiogenesis, CatK, as a member of the lysosomal cysteine and aspartic proteinase family, has been shown to be one of the most potent mammalian collagenases. It plays a vital role in maintaining the homeostasis of extracellular matrix.^33^ Pharmacological inhibition of CatK is a promising novel approach for postmenopausal osteoporosis therapy.^34^ Overexpression of CatK in mice decreases collagen deposition and lung resistance in response to bleomycin‐induced pulmonary fibrosis.^35^ We further reported that CatK has some effects on the metabolism of collagen in the late stage of MI. This might be the reason why CatK is mediating a protective effect in cardiac function.

Previous studies have reported that miR‐185 plays important roles in regulation of cell proliferation and apoptosis in response to stress such as oscillating glucose.^36^ As a tumour suppressive gene, miR‐185 is a pivotal mediator in the cellular response to hypoxia, a state that directly affects angiogenesis.^37^ In this study, we further uncovered the potential functions of miR‐185 as a suppressor of angiogenesis in revascularisation in ischaemic heart by regulating CatK gene expression. Moreover, the miR‐185‐5p/CatK axis is a key mechanism contributing to the recovery of cardiac function after MI.

We demonstrated that miR‐185‐5p was a key regulation of revascularisation in heart after ischaemia, and may act as a potential therapeutic target in patients with ACS. As an endothelial rich microRNA, miR‐185‐5p is positive in blood and circulating miR‐185 has been reported as a biomarker for clinical outcome in patients with dilated cardiomyopathy.^38^ By using high‐throughput RNA sequencing, we found 18 microRNAs including miR‐185‐5p in blood were differentially expressed in ACS patient. The reduction of circulating miR‐185‐5p in patients with ACS was further confirmed by quantitative PCR. In vivo experiments also indicate that infusion of miR‐185‐5p agomir delayed the recovery of heart function after MI. Combining the results, we thought that low level of miR‐185‐5p in blood may be associated with the good prognosis of ACS. Consistent with this notion, it has reported that circulating CatK, as a target of miR‐185‐5p identified by us, is a potential novel biomarker of coronary artery disease.^39^ How miR‐185‐5p is down‐regulated in endothelial cells by hypoxia and reduced in blood in ACS patient needs further investigation.

In summary, we identified that circulating miR‐185‐5p is substantially reduced in ACS patients. Endogenous inhibition of miR‐185‐5p in endothelial cells promotes angiogenesis and accelerates the repair of heart function after MI through targeting CatK gene expression (Figure S6D). Therefore, modulation of CatK gene expression by miR‐185‐5p could be effective for an angiogenic therapy in treating ischaemic diseases, including MI, stroke, peripheral artery disease and wound healing in diabetes.^40^

## DISCLOSURE

None.

## AUTHOR CONTRIBUTION

Y.P.B. conceived the experiments and wrote the manuscript; Y.P.B. and C.C.L. funded this study; X.T.Q. designed and conducted the majority of the experiments; X.T.Q. and C.C.L. performed statistical analysis and prepared all the figures; Q.S., J.P.Z., H.J.Y., W.Z.W. and L.F.H. performed the partial experiments; G.G.Z. and C.E.T. analysed the partial data.

## References

[1] Mukherjee D , Campbell CL . Optimal management of hypertension in patients with ischemic heart disease. Cardiovasc Hematol Agents Med Chem. 2009;7:198‐205. 19689258 10.2174/187152509789105435

[2] Landmesser U , Wollert KC , Drexler H . Potential novel pharmacological therapies for myocardial remodelling. Cardiovasc Res. 2009;81:519‐527. 19019834 10.1093/cvr/cvn317

[3] Ebrahimian TG , Heymes C , You D , et al. NADPH oxidase‐derived overproduction of reactive oxygen species impairs postischemic neovascularization in mice with type 1 diabetes. Am J Pathol. 2006;169:719‐728. 16877369 10.2353/ajpath.2006.060042 PMC1698801

[4] Tamarat R , Silvestre JS , Huijberts M , et al. Blockade of advanced glycation end‐product formation restores ischemia‐induced angiogenesis in diabetic mice. Proc Natl Acad Sci USA. 2003;100:8555‐8560. 12805564 10.1073/pnas.1236929100 PMC166267

[5] Rhoades MW , Reinhart BJ , Lim LP , Burge CB , Bartel B , Bartel DP . Prediction of plant microRNA targets. Cell. 2002;110:513‐520. 12202040 10.1016/s0092-8674(02)00863-2

[6] Njock MS , Fish JE . Endothelial miRNAs as cellular messengers in cardiometabolic diseases. Trends Endocrinol Metab. 2017;28:237‐246. 27989505 10.1016/j.tem.2016.11.009

[7] Jansen F , Yang X , Hoelscher M , et al. Endothelial microparticle‐mediated transfer of MicroRNA‐126 promotes vascular endothelial cell repair via SPRED1 and is abrogated in glucose‐damaged endothelial microparticles. Circulation. 2013;128:2026‐2038. 24014835 10.1161/CIRCULATIONAHA.113.001720

[8] Caporali A , Meloni M , Vollenkle C , et al. Deregulation of microRNA‐503 contributes to diabetes mellitus‐induced impairment of endothelial function and reparative angiogenesis after limb ischemia. Circulation. 2011;123:282‐291. 21220732 10.1161/CIRCULATIONAHA.110.952325

[9] Liang WJ , Zhou SN , Shan MR , et al. AMPKalpha inactivation destabilizes atherosclerotic plaque in streptozotocin‐induced diabetic mice through AP‐2alpha/miRNA‐124 axis. J Mol Med (Berl). 2018;96:403‐412. 29502204 10.1007/s00109-018-1627-8

[10] Zhu LP , Zhou JP , Zhang JX , et al. MiR‐15b‐5p regulates collateral artery formation by targeting AKT3 (protein kinase B‐3). Arterioscler Thromb Vasc Biol. 2017;37:957‐968. 28254819 10.1161/ATVBAHA.116.308905

[11] Bai YP , Zhang JX , Sun Q , et al. Induction of microRNA‐199 by nitric oxide in endothelial cells is required for nitrovasodilator resistance via targeting of prostaglandin I2 synthase. Circulation. 2018;138:397‐411. 29431644 10.1161/CIRCULATIONAHA.117.029206

[12] Li P , Yin YL , Guo T , et al. Inhibition of aberrant microRNA‐133a expression in endothelial cells by statin prevents endothelial dysfunction by targeting GTP cyclohydrolase 1 in vivo. Circulation. 2016;134:1752‐1765. 27765794 10.1161/CIRCULATIONAHA.116.017949 PMC5120771

[13] Chen Y , Gorski DH . Regulation of angiogenesis through a microRNA (miR‐130a) that down‐regulates antiangiogenic homeobox genes GAX and HOXA5. Blood. 2008;111:1217‐1226. 17957028 10.1182/blood-2007-07-104133 PMC2214763

[14] Hu L , Cheng XW , Song H , et al. Cathepsin K activity controls injury‐related vascular repair in mice. Hypertension. 2014;63:607‐615. 24343118 10.1161/HYPERTENSIONAHA.113.02141 PMC3945206

[15] Voellenkle C , Rooij J , Guffanti A , et al. Deep‐sequencing of endothelial cells exposed to hypoxia reveals the complexity of known and novel microRNAs. RNA. 2012;18:472‐484. 22282338 10.1261/rna.027615.111 PMC3285935

[16] Mitchell PS , Parkin RK , Kroh EM , et al. Circulating microRNAs as stable blood‐based markers for cancer detection. Proc Natl Acad Sci USA. 2008;105:10513‐10518. 18663219 10.1073/pnas.0804549105 PMC2492472

[17] Hou J , Liu L , Zhu Q , et al. MicroRNA‐185 inhibits angiogenesis in human microvascular endothelial cells through targeting stromal interaction molecule 1. Cell Biol Int. 2016;40:318‐328. 26694763 10.1002/cbin.10572

[18] Ho JJ , Metcalf JL , Yan MS , et al. Functional importance of Dicer protein in the adaptive cellular response to hypoxia. J Biol Chem. 2012;287:29003‐29020. 22745131 10.1074/jbc.M112.373365 PMC3436557

[19] Cohen RA , Murdoch CE , Watanabe Y , et al. Endothelial cell redox regulation of ischemic angiogenesis. J Cardiovasc Pharmacol. 2016;67:458‐464. 26927696 10.1097/FJC.0000000000000381 PMC4899292

[20] Xu MJ , Song P , Shirwany N , et al. Impaired expression of uncoupling protein 2 causes defective postischemic angiogenesis in mice deficient in AMP‐activated protein kinase alpha subunits. Arterioscler Thromb Vasc Biol. 2011;31:1757‐1765. 21597006 10.1161/ATVBAHA.111.227991 PMC3158995

[21] Iso T , Hamamori Y , Kedes L . Notch signaling in vascular development. Arterioscler Thromb Vasc Biol. 2003;23:543‐553. 12615665 10.1161/01.ATV.0000060892.81529.8F

[22] Limbourg FP , Takeshita K , Radtke F , Bronson RT , Chin MT , Liao JK . Essential role of endothelial Notch1 in angiogenesis. Circulation. 2005;111:1826‐1832. 15809373 10.1161/01.CIR.0000160870.93058.DD PMC2633594

[23] Zalc A , Hayashi S , Aurade F , et al. Antagonistic regulation of p57kip2 by Hes/Hey downstream of Notch signaling and muscle regulatory factors regulates skeletal muscle growth arrest. Development. 2014;141:2780‐2790. 25005473 10.1242/dev.110155

[24] Kobayashi T , Terada Y , Kuwana H , et al. Expression and function of the Delta‐1/Notch‐2/Hes‐1 pathway during experimental acute kidney injury. Kidney Int. 2008;73:1240‐1250. 18418349 10.1038/ki.2008.74

[25] Yu JG , Zhang EH , Liu AJ , Liu JG , Cai GJ , Su DF . Ketanserin improves cardiac performance after myocardial infarction in spontaneously hypertensive rats partially through restoration of baroreflex function. Acta Pharmacol Sin. 2013;34:1508‐1514. 24241347 10.1038/aps.2013.147 PMC4002568

[26] Lloyd PG , Yang HT , Terjung RL . Arteriogenesis and angiogenesis in rat ischemic hindlimb: role of nitric oxide. Am J Physiol Heart Circ Physiol. 2001;281:H2528‐H2538. 11709420 10.1152/ajpheart.2001.281.6.H2528

[27] Heil M , Eitenmuller I , Schmitz‐Rixen T , Schaper W . Arteriogenesis versus angiogenesis: similarities and differences. J Cell Mol Med. 2006;10:45‐55. 16563221 10.1111/j.1582-4934.2006.tb00290.x PMC3933101

[28] Belkaya S , Murray SE , Eitson JL , de la Morena MT , Forman JA , van Oers NS . Transgenic expression of microRNA‐185 causes a developmental arrest of T cells by targeting multiple genes including Mzb1. J Biol Chem. 2013;288:30752‐30762. 24014023 10.1074/jbc.M113.503532 PMC3798545

[29] Guo R , Hua Y , Rogers O , Brown TE , Ren J , Nair S . Cathepsin K knockout protects against cardiac dysfunction in diabetic mice. Sci Rep. 2017;7:8703. 28821796 10.1038/s41598-017-09037-z PMC5562704

[30] Hua Y , Zhang Y , Dolence J , Shi GP , Ren J , Nair S . Cathepsin K knockout mitigates high‐fat diet‐induced cardiac hypertrophy and contractile dysfunction. Diabetes. 2013;62:498‐509. 23069627 10.2337/db12-0350 PMC3554365

[31] Hua Y , Xu X , Shi GP , Chicco AJ , Ren J , Nair S . Cathepsin K knockout alleviates pressure overload‐induced cardiac hypertrophy. Hypertension. 2013;61:1184‐1192. 23529168 10.1161/HYPERTENSIONAHA.111.00947 PMC3929275

[32] Hua Y , Robinson TJ , Cao Y , Shi GP , Ren J , Nair S . Cathepsin K knockout alleviates aging‐induced cardiac dysfunction. Aging Cell. 2015;14:345‐351. 25692548 10.1111/acel.12276 PMC4406663

[33] Garnero P , Borel O , Byrjalsen I , et al. The collagenolytic activity of cathepsin K is unique among mammalian proteinases. J Biol Chem. 1998;273:32347‐32352. 9822715 10.1074/jbc.273.48.32347

[34] Mukherjee K , Chattopadhyay N . Pharmacological inhibition of cathepsin K: a promising novel approach for postmenopausal osteoporosis therapy. Biochem Pharmacol. 2016;117:10‐19. 27106079 10.1016/j.bcp.2016.04.010

[35] Srivastava M , Steinwede K , Kiviranta R , et al. Overexpression of cathepsin K in mice decreases collagen deposition and lung resistance in response to bleomycin‐induced pulmonary fibrosis. Respir Res. 2008;9:54. 18638383 10.1186/1465-9921-9-54 PMC2490691

[36] La Sala L , Cattaneo M , De Nigris V , et al. Oscillating glucose induces microRNA‐185 and impairs an efficient antioxidant response in human endothelial cells. Cardiovasc Diabetol. 2016;15:71. 27137793 10.1186/s12933-016-0390-9 PMC4852407

[37] Qu F , Cui X , Hong Y , et al. MicroRNA‐185 suppresses proliferation, invasion, migration, and tumorigenicity of human prostate cancer cells through targeting androgen receptor. Mol Cell Biochem. 2013;377:121‐130. 23417242 10.1007/s11010-013-1576-z

[38] Yu M , Liang W , Xie Y , et al. Circulating miR‐185 might be a novel biomarker for clinical outcome in patients with dilated cardiomyopathy. Sci Rep. 2016;6:33580. 27645404 10.1038/srep33580 PMC5028782

[39] Cheng XW , Kikuchi R , Ishii H , et al. Circulating cathepsin K as a potential novel biomarker of coronary artery disease. Atherosclerosis. 2013;228:211‐216. 23369704 10.1016/j.atherosclerosis.2013.01.004

[40] Bednarska J , Bednarska‐Chabowska D , Adamiec‐Mroczek J . Coronary artery disease: new insights into revascularization treatment of diabetic patients. Adv Clin Exp Med. 2017;26:1163‐1167. 29211367 10.17219/acem/68980

