# Supplementary material for: Endogenous reduction of miR‐185 accelerates cardiac function recovery in mice following myocardial infarction via targeting of cathepsin K
Source: J Cell Mol Med. 2018 Nov 18;23(2):1164–73. doi: 10.1111/jcmm.14016 (PMC6349160; doi:10.1111/jcmm.14016)
Supplement: Supplementary file 1 [file JCMM-23-1164-s001.docx]

**Online Supplements**

**Endogenous reduction of miR-185 accelerates** **cardiac function recovery in mice following myocardial infarction via targeting of cathepsin K**

**Running title:** miR-185 in angiogenesis

Chuan-Chang Li^1,3,a^, Xue-Ting Qiu^1,a^, Quan Sun^2^, Ji-Peng Zhou^3^, Hui-Jun Yang^2^, Wan-Zhou Wu^1^, Ling-Fang He^1^, Can-E Tang^4^, Guo-Gang Zhang^2,3^, Yong-Ping Bai^1,3,b^

^1^Department of Geriatric Medicine, Xiangya Hospital, Central South University, Changsha, China 410008; ^2^Department of Cardiovascular Medicine, Xiangya Hospital, Central South University, Changsha, China 410008; ^3^National Clinical Research Center for Geriatric Disorder, Xiangya Hospital, Central South University, Changsha, China 410008; ^4^Institute of Medical Science Research, Xiangya Hospital, Central South University, Changsha, China 410008

^a^ Chuan-Chang Li and Xue-Ting Qiu contributed equally to this work.

^b^ Correspondence to Yong-Ping Bai, No 87, Xiangya Road, Changsha, China, 410008. E-mail baiyongping@csu.edu.cn.

**Contents**

1. Supplementary Materials and Methods

2. Supplementary Figure S1-S8

3. Supplementary Table S1-S3

**Supplementary Materials and Methods**

**Materials**

Adenovirus of cathepsin K (CatK) cDNA, scramble shRNA, and CatK shRNA were purchased from ORIGENE Technologies (Beijing, China). Primary antibodies against CatK, GAPDH, CD31, c-notch1, t-notch1, hypoxia inducible factor 1 alpha (HIF-1α), alpha smooth muscle actin (α-SMA), and secondary antibodies were obtained from Cell Signaling Company or Santa Cruz Company. miR-185-5p mimic, negative mimic, inhibitor and negative inhibitor were purchased from Ribobio technologies (Guangzhou, China). The concentration and specific description were detailedly presented in Methods. The primer sets used were presented in Supplemental Table S4.

**Patient recruitments**

Patients who underwent coronary angiography were enrolled from the cardiac catheterization room of Xiangya Hospital, Central South University, Hunan Province, China during 2014 and 2016. Thirty patients with acute coronary syndrome (ACS) were selected with previously typical unstable angina pectoris or diagnosed as myocardial infarction (MI). A significant stenosis was defined as diameter stenosis ≥ 90% through coronary angiography. The patients were excluded if they had other potentiality that could influence neovascularization, like symptomatic peripheral arterial disease, decompensated heart failure, any concomitant inflammation or infectious diseases, neoplastic diseases, and severe liver and kidney dysfunction. 30 patients who had no angina pectoris and coronary angiography revealed stenosis as diameter stenosis < 50% were chosen as control. We documented all the cardiac history and risk factors. The demographic data were presented in Supplemental Table S1. Informed consent was obtained from all participants. The study protocol was approved by the Ethics Committee of Xiangya Hospital, Central South University, Changsha, Hunan, China.

**High throughput RNA sequencing**

Total RNA from patient’s blood was used to prepare the miRNA sequencing library and high-throughput RNA sequencing was performed by the Kang Cheng Company of China in Shanghai as described previously^[1]^. Briefly, an Agilent 2100 Bioanalyzer was used to quantify the libraries, and the samples were then diluted to a final concentration of 8 pM. Next, cluster generation was performed on the Illumina cBot using the TruSeq Rapid SR cluster kit (#GD-402-4001, Illumina), following the manufacturer’s instructions. The DNA fragments in the libraries were denatured with 0.1 M NaOH to generate single­stranded DNA molecules. They were then observed on Illumina flow cells, amplified in situ, and sequenced for 36 cycles with an Illumina HiSeq 2000 (Illumina, San Diego, CA, USA), according to the manufacturer’s instructions.

**Induction of myocardial infarction (MI) in mice**

Male mice (8-12 weeks old, 25 ± 5 g) were purchased from Hua-Fu-Kang Animal Company (Beijing, China). All animals were housed in temperature-controlled cages with a 12-hour light-dark cycle. This study was carried out in strict accordance with the recommendations in the Guide for the Care and Use of Laboratory Animals of the National Institutes of Health. The animal protocol was reviewed and approved by the Animal Care and Use Committee of Central South University.

A minimally invasive approach established by us was used to induce MI in mice without thoracotomy as described previously^[2]^. Briefly, once anesthetized, two small incisions (0.5 cm long) are made on the left and right chest skin with the scissors to expose the 3rd intercostal space. Echocardiography is performed using a VEVO 2100 imaging system (Visual Sonics Inc., Toronto, Canada) with a 30 MHz phased array transducer and a frame rate of 235/s. The echocardiography probe (MS-400) is placed perpendicular to the sagittal plane of the chest within the 3rd intercostal space, imaging the left ventricle (LV) short axis. A small straight needle (0.2 mm in diameter) was inserted at the costal angle of the superior margin of the 3^rd^ rib in the left chest. Under the guide of ultrasound, the heart is punctured in the inferior of LCA by a 8-0 silk suture attached to the needle. The needle is coming out of skin from the right chest. Then, the needle is inserted back from the right to of the left. When the needle passes through the heart, it goes through above LCA under ultrasound and came out the skin from the same site in the left chest. Once a loose knot is made, the needle is inserted back from the left to the right in the chest. The LCA is now located inside of the knot. Ligation of LCA by pulling the two ends of the suture carefully. The ischemia was confirmed by the elevation of ST segment recorded by the echocardiography imaging system during the surgery.

**Animal protocols**

The animal experiments were divided into two parts. In the first part of the animal study, as shown in Supplementary Figure S5A, C57B16 mice received negative or miR-185-5p agomir administration through Alzet osmotic minipumps for 5 weeks (80 mg/kg/day)^[3]^. MiR-185-5p agomir (5’-UGGAGAGAAAGGCAGUUCCUGA-3’) was synthesized by Ribobio (Guangzhou, China). A negative agomir (5’-UUUGUACUACACAAAAGUACUG-3’) was synthesized and used as control. MI surgery was performed at one week after agomir infusion. After 28 postoperative days, mice were subjected to heart function detection by echocardiography and then to be sacrificed under anaesthesia. Heart was harvested for analysis of fluorescence in situ hybridization (FISH), immunohistochemistry (IHC), immunofluorescence (IFC), real time reverse transcription PCR (qPCR), masson’s trichrome staining, or western blot.

In the second part of the animal study (Supplementary Figure S5C), mice were infected with adenovirus vector or expressing CatK shRNA via tail vein injection every other week. For *in vivo* infection, adenovirus were injected via tail vein in 100 µl of PBS containing 7.6 X 10^9^ PFUs of loaded adenovirus per mouse as described previously^[4]^. MI surgery was performed at two weeks after virus infection. After 28 postoperative days, mice were subjected to heart function detection by echocardiography and then to be sacrificed under anaesthesia. Heart was harvested for analysis of FISH, IHC, IFC, PCR, masson’s trichrome staining, or western blot.

**Detection of miR-185-5p by FISH**

As described previously with minor modifications^[5, 6]^, FISH of miR-185-5p was performed on clips or 5-μm paraffin embedded arterial sections. Briefly, paraffin embedded tissue sections were cut using a microtome (Leica, RM2235) and mounted on polylysine microscope slides (Fisher Scientific) and then stored at room temperature (RT) until FISH. Paraffin wax was removed in xylene, sections rehydrated in a series of decreasing ethanol solutions and washed with PBS before fixing in 4% paraformaldehyde. To block endogenous peroxide activity, tissue sections were treated with 0.3% H_2_O_2_ and washed in PBS before acetylating in acetic anhydride/triethanolamine. Sections were then washed in 2 X SSC and PBS before permeabilisation with proteinase K (5 μg/ml) and washes with PBS. Probes (5’ & 3’-DIG labelled LNA miRCURY probes; Exiqon) were denatured at 90°C before dilution inhybridisation buffer (50% formamide, 0.3 M NaCl, 20 mM Tris-HCL, 5 mM EDTA, 10 mM NaPO_4_, 10% Dextran sulphate, 1 X Denhardt’s solution, 0.5 mg/ml yeast tRNA). Tissue sections were hybridised with miR-185-5p (200 nM) overnight at 21°C below the predicted Tm value of the probe. After post-hybridization washes in 5 X SSC at RT; 50% formamide/1 X SSC/0.1% Tween 20 at the hybridisation temperature; 0.2 X SSC at RT, FISH signals were detected using an Anti-Digoxigenin antibody and the tyramide signal amplification system (PerkinElmer) according to the manufacturer's instructions. Tissue sections were mounted in Vectashield (Vector laboratories). All fluorescence images were analyzed with a Nikon TE2000-U inverted microscope.

**Determination of miR-185-5p by Real time reverse transcription PCR (qPCR)**

Total RNA was isolated from plasma, cells or tissues according to the manufacturer's protocol with the mirVana PARIS Kit (Ambion, England) from the plasma of the validation cohort. The miRNA from each sample was quantified by SYBR Premix Ex Taq qPCR assays (TaKaRa, Japan). Real-time PCR was performed on an ABI 7500 real­time PCR system, and amplification efficiencies were checked by standard curves. The miRNA expression levels of miR-185-5p were normalized to cel-miR­238­3p (5’- uuuguacuccgaugccauucaga-3’) expression.

**RNA Quantification by real-time RT-PCR**

Total RNA was isolated using a TRIzol-based (Invitrogen, USA) RNA isolation protocol. RNA concentration was quantitated by applying the Nano Drop ND-100 Spectrophotometer (Nano Drop Technologies, Wilmington, DE). For detections of CatK, Hes1, Hey1, Hey2 and GAPDH, cDNAs were synthesized by using PrimeScript TM RT reagent kit (TaKaRa, Japan) according to the manufacturer’s instructions. Real-time PCRs were performed by using SYBR Premix Ex Taq qRT-PCR assays under ABI 7500 real-time PCR system (Life Technologies, USA). GAPDH were used as internal standard to normalize the mRNA expression level using 2-ΔΔCt method.

**Echocardiography**

Mice were anesthetized with 1.5-2% isoflurane and then evaluated by Vevo 2100 echocardiography (VisualSonics Inc, Toronto, Ontario, Canada) with a 30 MHz central frequency scan head to detect cardiac function. The following parameters were measured from M-mode images taken from the parasternal short-axis view at papillary muscle level: left ventricular fractional shortening (FS) and left ventricular ejection fraction (EF), the left ventricle internal diameter and left ventricle volume in diastole or systole. At least three measurements were obtained and averaged for each mouse.

**Masson’s trichrome staining**

Heart samples were fixed in 4% PFA and then embedded in paraffin. 4-μm thick sections were subjected to Masson’s trichrome staining following a standard procedure. Images of the left ventricular area of each section were taken by Nikon model with Spot Insight camera. Image J Software was used to quantify fibrotic region in each section.

**Immunohistochemistry (IHC) and immunofluorescence (IFC)**

Histological analysis was performed on perfusion/fixed hearts collected from mice at 28 postoperative days after surgery. Briefly, mice were euthanized by pentobarbitol sodium (1%, 50 mg/kg). The chest was opened and the heart was arrested in diastole by intraventricular injection of 10% KCl. The myocardial vasculature was perfused, followed by 10 min perfusion with 4% PFA. The hearts were harvested and fixed in 4% PFA for 24 hours. The formalin‑fixed tissues were embedded in paraffin wax and cut into 4-µm sections. For the measurement of capillary density, we applied IHC analysis of CD31 antibody (1:200 dilution), IFC analysis of CD31 antibody (1:50 dilution), Catk (1:100 dilution) and ɑ-SMA (1:100 dilution). Transverse sections of the short axis of heart for each sample were used in this analysis. Five fields on the slide were randomly selected at X400 magnification and analyzed by image J.

**Cell cultures**

Human umbilical vein endothelial cells (HUVECs) were originally purchased from ATCC and cultured according to standard procedures. Cells were cultivated in DMEM medium (gibco, life technologies, USA) supplement with 10% fetal bovine serum (FBS), 100 U/ml penicillin, and 100 μg/mlstreptomycin. The medium was refreshed every other day. Either miR-185-5p mimic (50nM), negative mimic (50nM), miR-185-5p inhibitor (100nM) and control inhibitor (100nM) were transfected into HUVECs for 24-48 hours using lipofectamine 2000 (Invitrogen, USA) according to the manufacturer's instructions. Then cells were maintained under hypoxia by a tri-gas incubator (37°C, 1% O_2_,5% CO_2_) for detected times.

**Luciferase reporter assay**

The miR-185-5p binding site in 3’-UTR of CatK mRNA was amplified by PCR from genomic DNA of 293T cells. The PCR products were gel purified, digested and inserted into the pMIR-RB-REPORT™ vector (Ribobio, China) to generate the reconstituted plasmid. The miR-185-5p binding site mutations were introduced using the Multisite-Quickchange (Stratagene, USA) according to the manufacturer's protocol and cloned into the pMIR-RB-REPORTTM vector. All inserted or mutated sequences were confirmed by DNA sequencing. Dual luciferase assay was used to quantify the effects of miR-185-5p interaction with the 3′-UTR of CatK mRNA. In all experiments, transfection efficiencies were normalized to hLuc luciferase that was constitutively expressed by pMIR-RB-REPORT™ vector.

**Western blot**

As described previously^[7]^, Cells or hearts were lysed for 30 min at 4°C in a lysis buffer. Total protein concentration was determined using bicinchoninic acid reagent. Total protein (20 μg) was resolved by SDS-polyacrylamide gel electrophoresis, transferred to a nitrocellulose membrane, and subjected to immunoblot analysis. The primary antibodies for CatK (1:200 dilution), notch1 (1:1000 dilution), c-notch1 (1:1000 dilution), GADPH (1:10000 dilution) and horseradish peroxidase­conjugated secondary antibody (1:5000 dilution) were used. The bands were visualized using enhanced chemiluminescence reagents and analysed with Image J.

**Cell proliferations**

Cell proliferations were determined by CCK-8 and BrdU assays as descried previously^[8]^. Cells were seeded (5 X 10^3^ cells/well) on 96-well plates for CCK-8 and on 24-well plates for BrdU assay. 10 μl of CCK­8 solution was added to each well and incubated for 2 hours. The absorbance of the plate was measured at 450 nm with a microplate reader (Molecular Devices, Menlo Park, CA). For BrdU assay, the 24-well plates were refleshed using cell culture medium containing BrdU (10 µM, Abcam) and then incubated for 2 hours. By removing the BrdU labeling solution from the cells and washed twice in PBS, cells were fixed before proceeding with immunostaining according to the manufacturer's instructions.

**Transwell migration assay**

As described previously, cells were starved overnight, trypsinized and resuspended in serum-free medium. 1X10^5^ cells were seeded in upper chamber of the Falcon Cell Culture Inserts (8.0 µm pore size; Corning 353097). Culture medium supplemented with 20% FBS was added to the lower chamber. Twenty-four hours later, non-migrated cells in upper chamber were removed by a cotton swab, and cells passed through the pores and attached on the lower surface of the membrane were fixed with methanol and stained with crystal violet. Ten fields of each cell group were snapped under an Olympus IX81 microscope with an Olympus IX-TVAD camera and the numbers of migrated cells were counted.

**Wound healing assay**

Cells were seeded into two separated chambers generated by the Culture-Inserts (Ibidi 80206) and allowed to grow into confluency overnight. The Culture-Inserts were gently removed by using sterile tweezers and then a cell-free gap (“wound”) of about 500 μm was so formed. Three wounds were generated for each cell group. All cells were cultured in serum-free medium thereafter and images were captured at 0, 24, and 48 hours. Area percentage of healing was analyzed by Image J software.

**Tube formation assay**

As described previously^[9]^, the formation of vessel-like structures by HUVECs on growth factor-reduced Matrigel (BD Biosciences) was performed as previously described. Briefly, 35-mm culture dishes were coated with Matrigel according to the manufacturer's instructions. The indicated cells were seeded on coated dishes at 3 X 10^5^ cells/dish in medium containing vascular endothelial growth factor (50 ng/mL) and incubated at 37°C for 8 hours under normoxic or hypoxic conditions. Tube formation was observed using an inverted phase contrast microscope (Nikon, Tokyo, Japan). Images were captured with a videographic system (DEI-750 CE Digital Output camera; Optronics, Goleta, CA). The degree of tube formation was quantified by measuring the tube length per fields (X 20) from each dish using the National Institutes of Health Image program.

**Statistical analysis**

All quantitative results are expressed as mean ± s.e.m. Comparisons between two groups were analyzed by unpaired Student's *t* test between two groups. Multiple comparisons over two groups were analyzed with a one-way ANOVA followed by Tukey *post-hoc* tests or Bonferroni *post-hoc* analyses. Categorical variables were compared by the chi-square test. Statistical analysis was conducted using IBM SPSS statistics 20.0 (IBM Corp., Armonk, NY, USA) and *P*<0.05 were considered as statistical significance.

**References**

[1]  **Voellenkle C, Rooij J, Guffanti A, Brini E, Fasanaro P, Isaia E, Croft L,**

**David M, Capogrossi MC, Moles A, Felsani A and Martelli F.** Deep-sequencing of endothelial cells exposed to hypoxia reveals the complexity of known and novel microRNAs. RNA. 2012; 18: 472-84.

[2] **Gao E, Lei Y H, Shang X, Huang ZM, Zuo L, Boucher M, Fan Q, Chuprun JK, Ma XL and Koch WJ.** A novel and efficient model of coronary artery ligation and myocardial infarction in the mouse. Circ Res. 2010; 107: 1445-53.

[3]  **Li H, Xie H, Liu W, Hu R, Huang B, Tan YF, Liao EY, Xu K, Sheng ZF, Zhou HD, Wu XP and Luo XH.** A novel microRNA targeting HDAC5 regulates osteoblast differentiation in mice and contributes to primary osteoporosis in humans. J Clin Invest. 2009; 119 :3666-77.

[4] **Hiltunen M O, Laitinen M, Turunen M P, Jeltsch M, Hartikainen J, Rissanen TT, Laukkanen J, Niemi M, Kossila M, Hakkinen TP, Kivela A, Enholm B, Mansukoski H, Turunen AM, Alitalo K, Yla-Herttuala S.** Intravascular adenovirus-mediated VEGF-C gene transfer reduces neointima formation in balloon-denuded rabbit aorta. Circulation. 2000; 102: 2262-68.

[5]  **Torella D, Iaconetti C, Catalucci D, Ellison GM, Leone A, Waring CD, BochicchioA, Vicinanza C, Aquila I, Curcio A, Condorelli G and Indolfi C.** MicroRNA-133 controls vascular smooth muscle cell phenotypic switch in vitro and vascular remodeling in vivo. Circ Res. 2011; 109: 880-93.

[6] **Li P, Yin YL, Guo T, Sun XY, Ma H, Zhu ML, Zhao FR, Xu P, Chen Y, Wan GR, Jiang F, Peng QS, Liu C, Liu LY and Wang SX.** Inhibition of Aberrant MicroRNA-133a Expression in Endothelial Cells by Statin Prevents Endothelial Dysfunction by Targeting GTP Cyclohydrolase 1 in Vivo. Circulation. 2016; 134: 1752-65.

[7]  **Bai YP, Xiao S, Tang YB, Tan Z, Tang H, Ren Z, Zeng H and Yang Z.** Shear stress-mediated upregulation of GTP cyclohydrolase/tetrahydrobiopterin pathway ameliorates hypertension-related decline in reendothelialization capacity of endothelial progenitor cells. J Hypertens. 2017; 35: 784-97.

[8] **Yang XH, Li P, Yin YL, Tu JH, Dai W, Liu LY and Wang SX.** Rosiglitazone via PPARgamma-dependent suppression of oxidative stress attenuates endothelial dysfunction in rats fed homocysteine thiolactone. J Cell Mol Med. 2015; 19: 826-35.

[9]  **Xu MJ, Song P, Shirwany N, Liang B, Xing J, Viollet B, Wang X, Zhu Y and Zou MH.** Impaired expression of uncoupling protein 2 causes defective postischemic angiogenesis in mice deficient in AMP-activated protein kinase alpha subunits. Arterioscler Thromb Vasc Biol. 2011; 31: 1757-65.

**
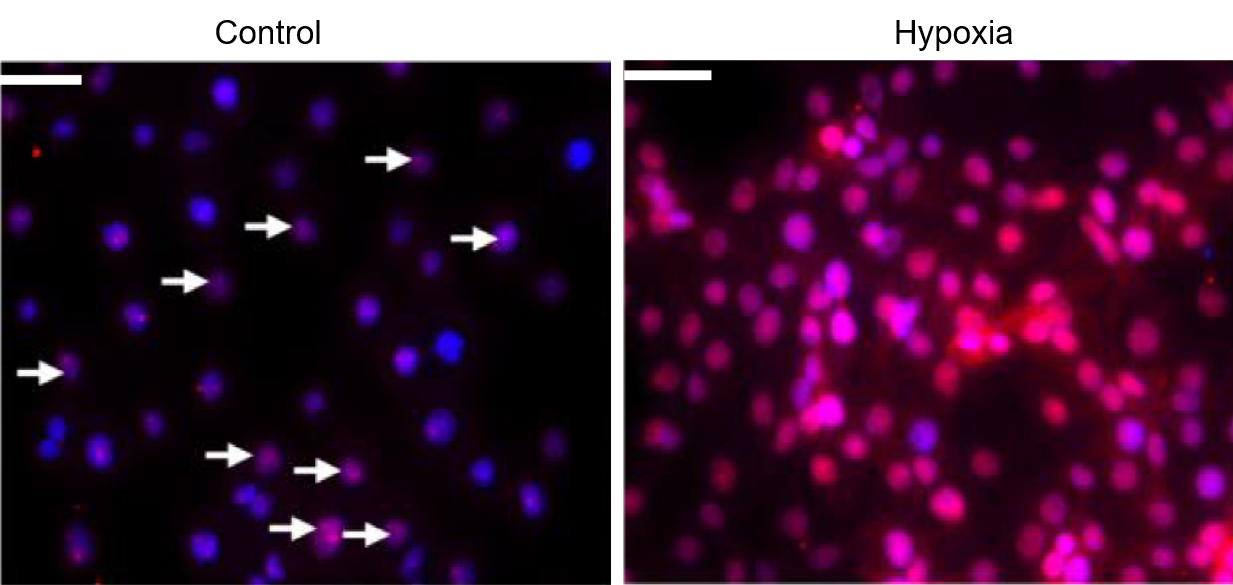
**

**Supplemental Figure S1. Hypoxia induces the expression of HIF-1α in HUVECs**. Cultured HUVECs were exposed to hypoxia for 24 hours. HIF-1α was determined by IFC. Representative photomicrographs were shown from 5 independent experiments. Red, HIF-1α. Blue, DAPI. Scar bar is 50 μm.

**
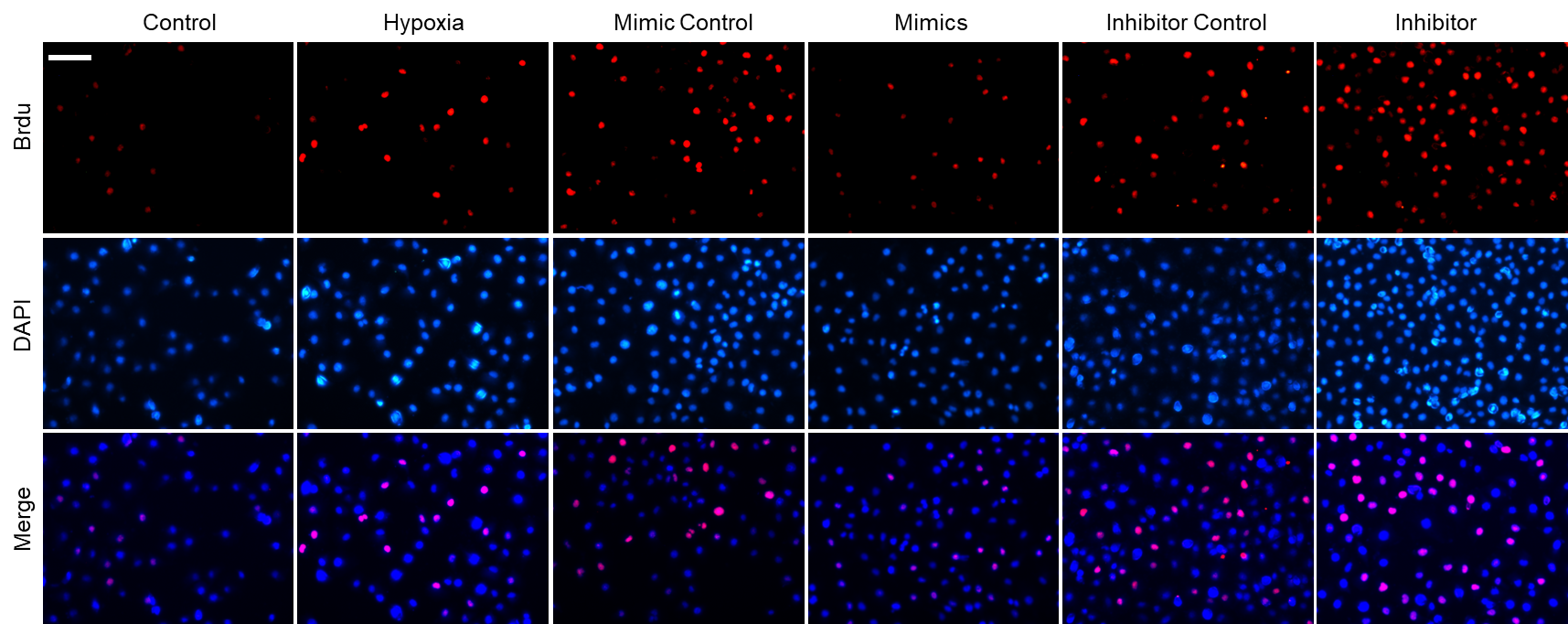
**

**Supplemental Figure S2. MiR-185-5p regulates cell proliferations in HUVECs under hypoxia**. Cultured HUVECs were transfected with miR-185-5p mimics (50 nM) or miR-185-5p inhibitors (100 nM) for 24 hours following hypoxia. Cell proliferations were determined by BrdU and representative labeling photomicrographs were shown from 5 independent experiments. Red, BrdU. Blue, DAPI. Scar bar is 50 μm.

**
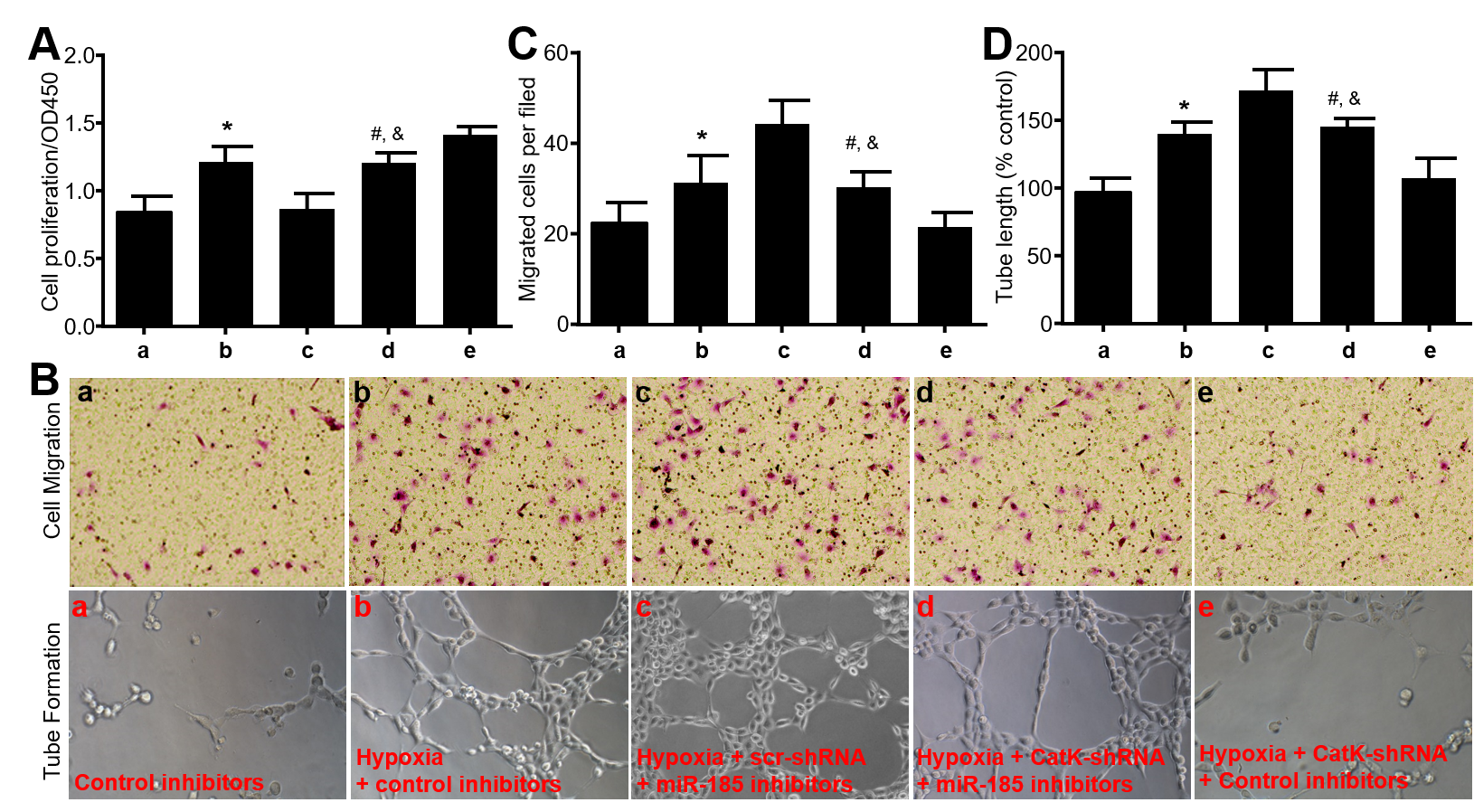
**

**Supplemental Figure S3. Silencing cathepsin K (CatK) bypasses the enhancive**

**effects of miR-185-5p inhibition on proliferation, migration, and tube formation of HUVECs under hypoxia.** Cultured HUVECs were infected with adenovirus expressing scramble shRNA or CatK shRNA for 48 hours followed by transfection with controls and miR-185-5p inhibitors (100 nM) for 24 hours. Then cells were exposed to hypoxia for 24 hours. a, Control inhibitors. b, Hypoxia plus control inhibitors. c, Hypoxia plus miR-185-5p inhibitors plus scramble shRNA. d, Hypoxia plus miR-185-5p inhibitors plus CatK shRNA. e, Hypoxia plus control inhibitors plus CatK shRNA. (**A**) Cell proliferations were determined by CCK8. (**B**) Cell migration and tube formation were determined in HUVECs after treatments. (**C** and **D**) Quantitative analysis of cell migration in **D** and tube formation in **E** were performed in data from **B**. N = 5 per group. **P* < 0.05 *vs* Control inhibitors (a). ^#^*P* < 0.05 *vs* Hypoxia plus miR-185-5p inhibitors plus scramble shRNA (c). ^&^*P* < 0.05 *vs* Hypoxia plus control inhibitors plus CatK shRNA (e).

**
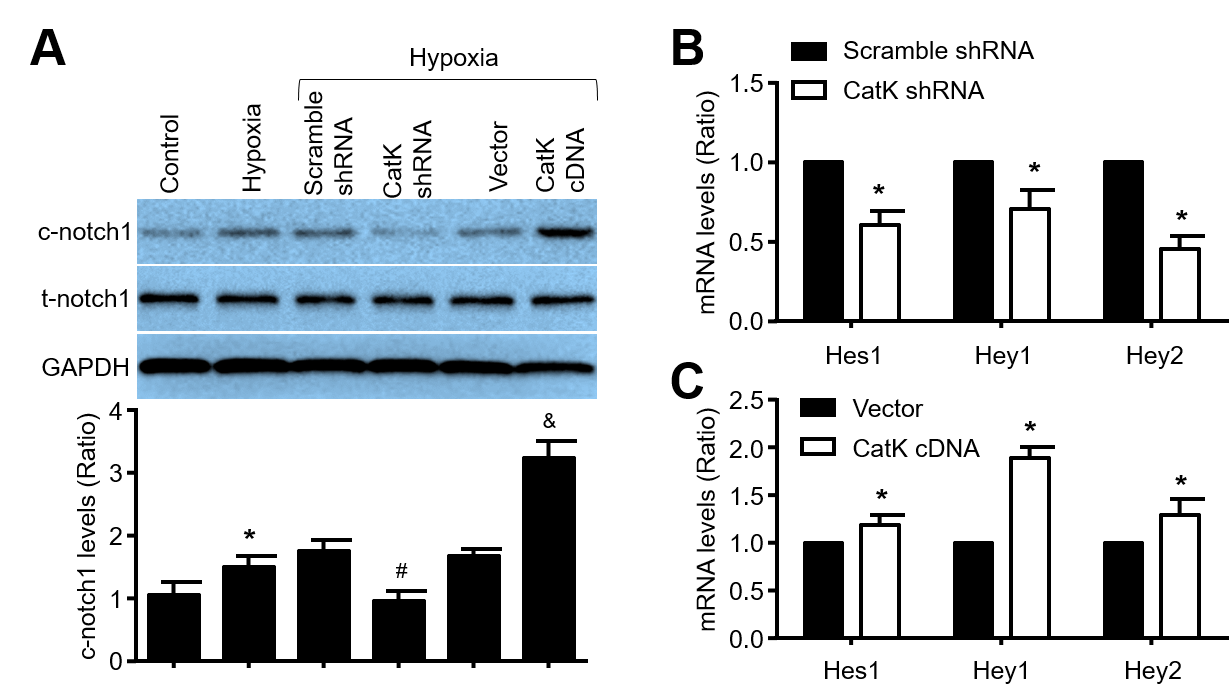
Supplemental Figure S4. Cathepsin K (CatK) regulates c-notch1, Hes1, Hey1 and Hey2 mRNA expressions in HUVECs.** Cultured HUVECs were infected with adenovirus harboring CatK shRNA or cDNA for 48 hours followed by hypoxia. (**A**) Total cell lysates were subjected to c-notch1 and t-notch1 by western blot. N = 5 per group. **P* < 0.05 *vs* Control. ^#^*P* < 0.05 *vs* Hypoxia plus scramble shRNA. ^&^*P* < 0.05 *vs* Hypoxia plus vector (e). (**B** and **C**) Quantification of Hes1, Hey1 and Hey2 mRNA in HUVECs by real-time RT-PCR. N = 5 per group. **P* < 0.05 *vs* Scramble shRNA or vector.

**
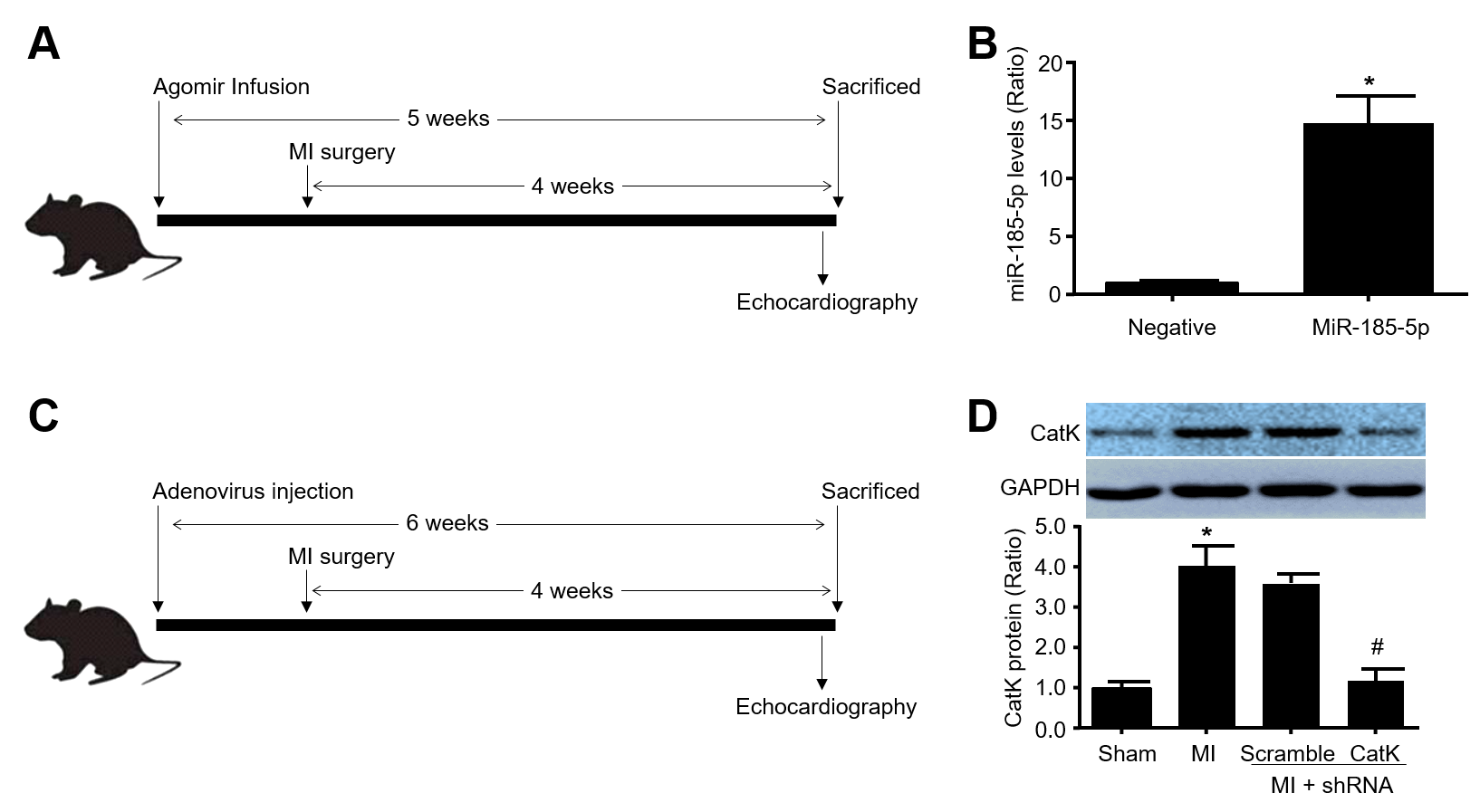
Supplemental Figure S5. Protocols of animal experiments and *in vivo* efficacy of miR-185-5p agomir and cathepsin K (CatK) gene silencing**. (**A**) The protocol of gain-function of miR-185-5p and MI model. (**B**) The expression of miR-185-5p was assessed by qPCR. N = 10-15 per group. **P* < 0.05 *vs* Negative agomir. (**C**) The protocol of loss-function of CatK and MI model. (**D**) The protein level of CatK was assayed by western blot. N = 10-15 per group. **P* < 0.05 *vs* Sham. ^#^*P* < 0.05 *vs* MI plus scramble shRNA.

**
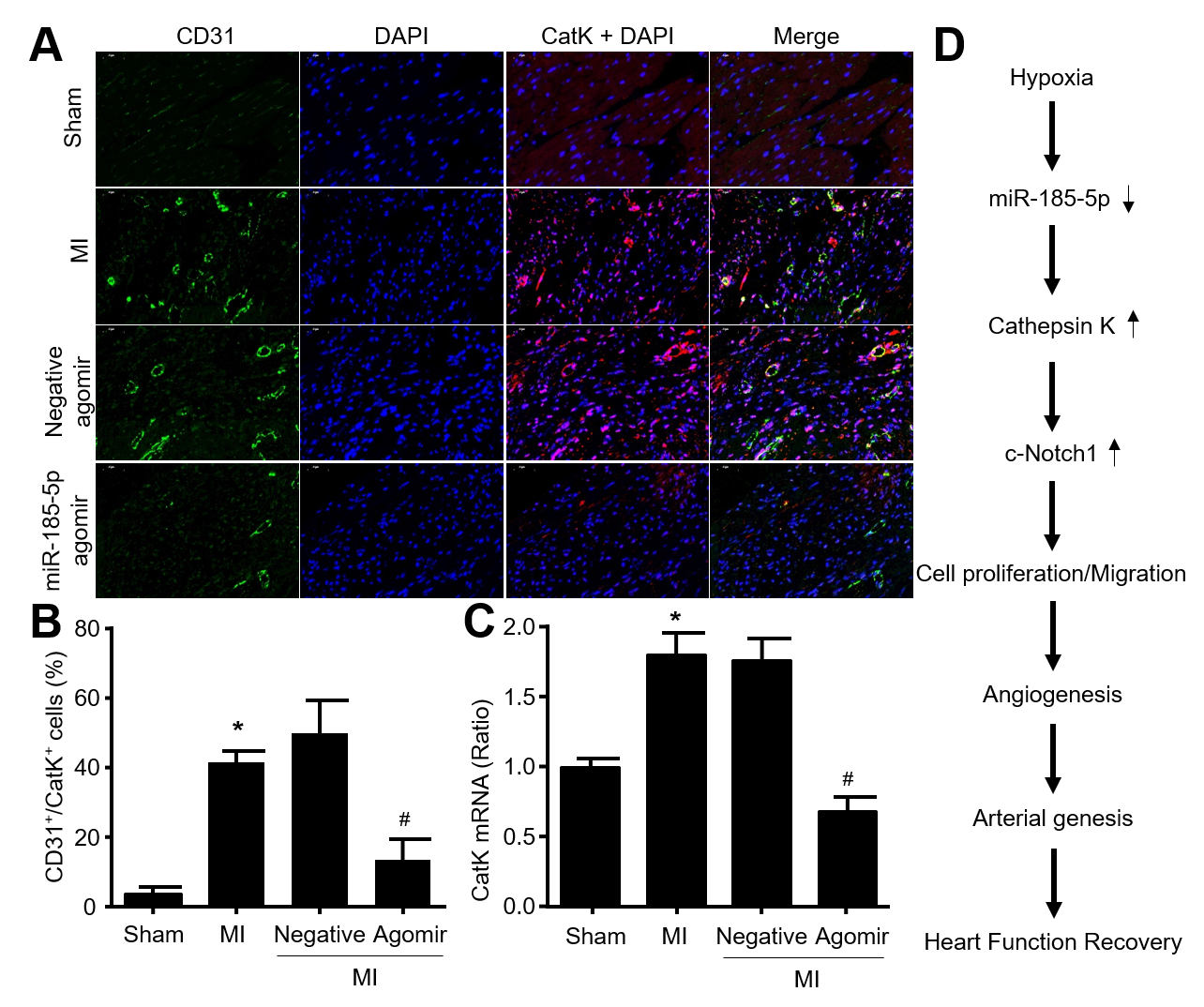
**

**Supplemental Figure S6. MiR-185-5p agomir reduces cathepsin K (CatK) gene expression in endothelial cells in hearts isolated from mice following MI**. The animal protocols of miR-185-5p agomir infusion and MI model induction were shown in Supplementary Figure S5A. (**A**) The CD31^+^/CatK^+^ cells in cross-sections of left ventricle in mice after 28 postoperative days were determined by performing IFC analysis of CD31 and CatK. Representative pictures were shown. (**B**) Number of CD31^+^/CatK^+^ cell was calculated in pictures from **A**. (**C**) CatK mRNA in heart was measured by real-time RT-PCR. N = 10-15 per group. **P* < 0.05 *vs* Sham. ^#^*P* < 0.05 *vs* MI plus Negative. (**D**) Proposed mechanism of angiogenesis in heart following MI.

**
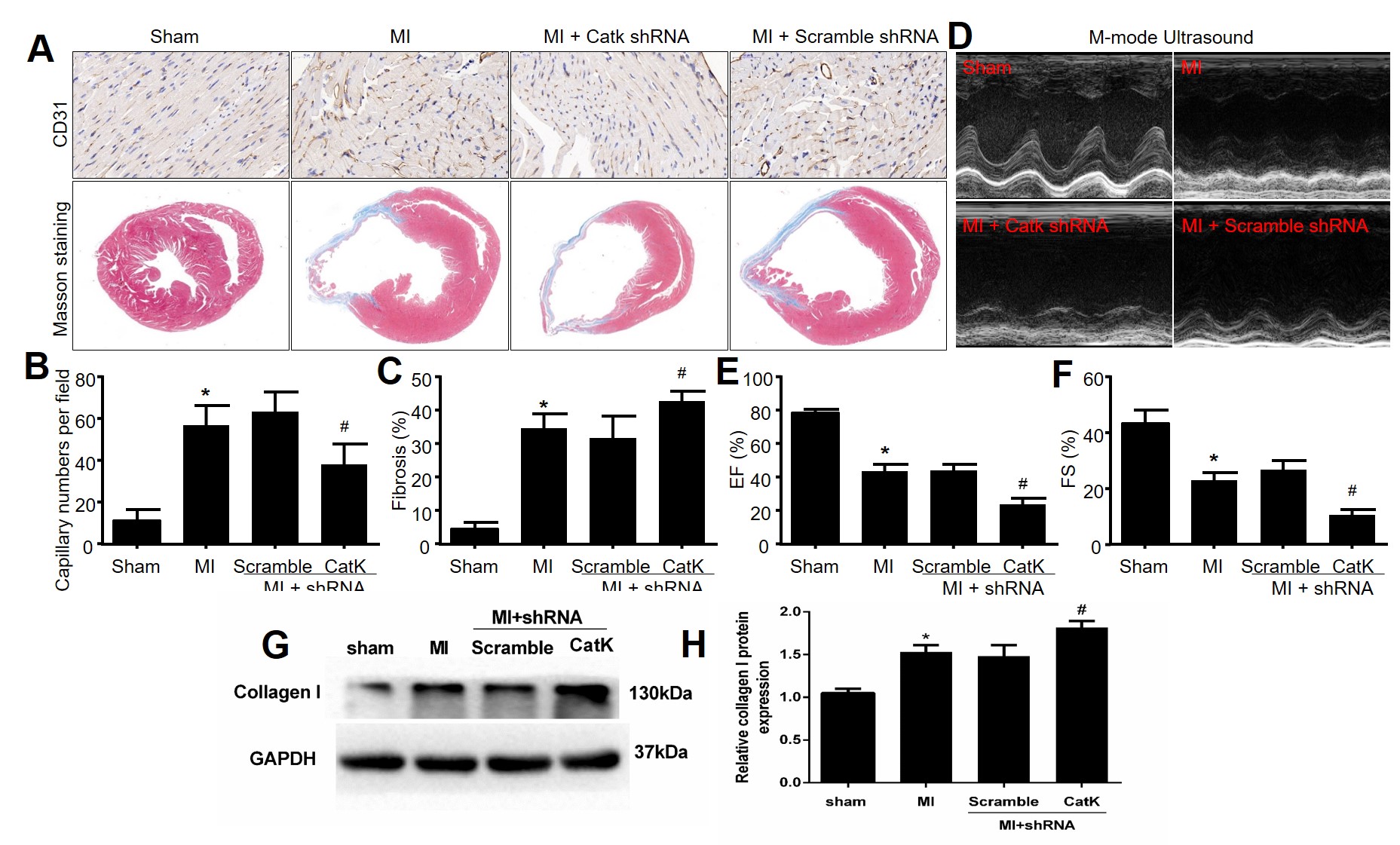
**

**Supplemental Figure S7. Knockdown of cathepsin K (CatK) impairs angiogenesis and the recovery of heart functions in mice following MI**. The protocol of loss-function of CatK and MI model was shown in Supplementary Figure S5C. (**A**-**C**) IHC analysis of CD31 and masson’s trichrome staining of fibrosis were performed in cross-sections of left ventricle in mice after 28 postoperative days. Representative pictures were presented in **A**. Quantitation of capillary intensities in **B** or fibrosis in **C** was conducted. (**D**-**F**) Heart functions were determined by echocardiography in mice after 28 postoperative days before scarified. Representative images of M-mode echocardiography were shown in **D**. Both EF in **E** and FS in **F** were calculated. (**G**-**H**) The protein level of collagen I were assayed by western blot in left ventricle after 28 postoperative days. Representative pictures were presented in **G**. Quantitation of relative collagen I protein expression in **H**. N = 10-15 per group. **P* < 0.05 *vs* Sham. ^#^*P* < 0.05 *vs* MI plus scramble shRNA.

**
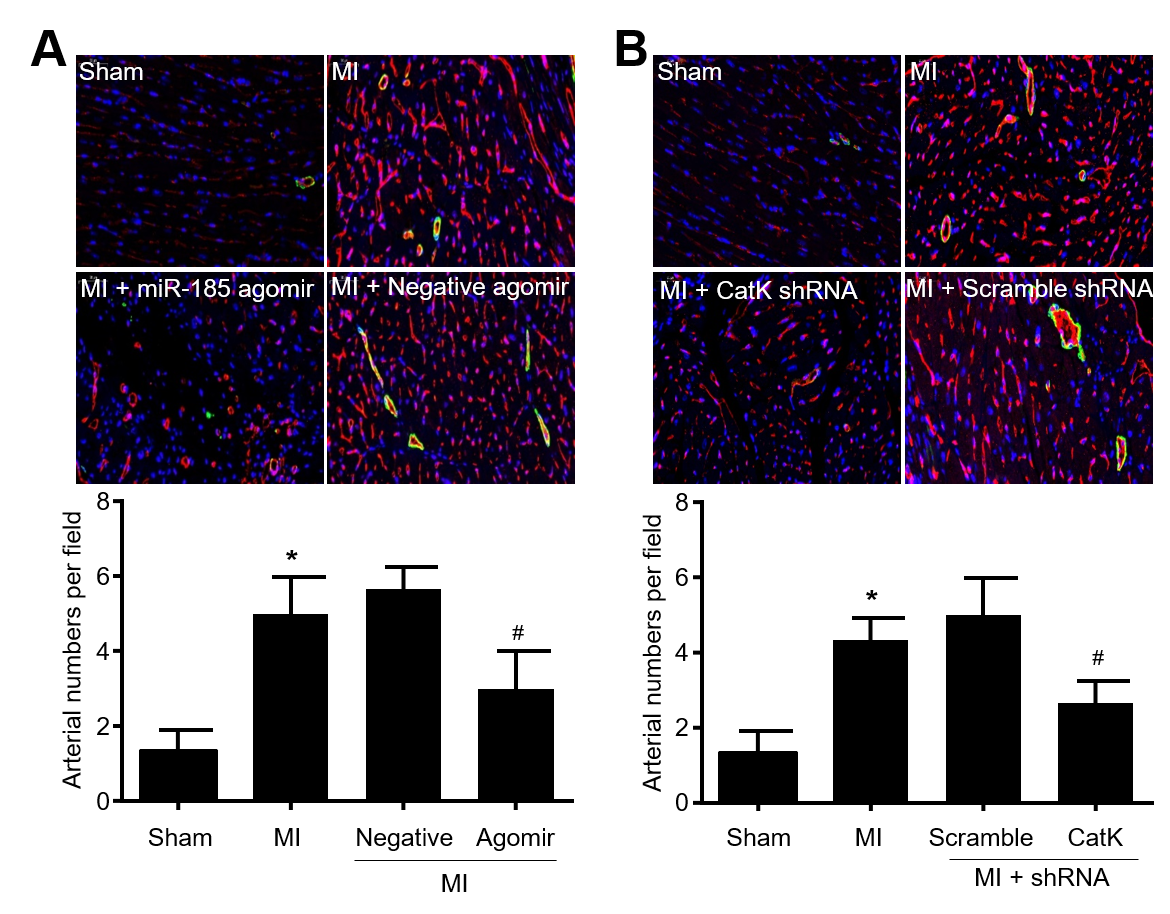
**

**Supplemental Figure S8. MiR-185-5p agonist and cathepsin K (CatK) inhibition suppress ischemia-induced arteriogenesis in mice with MI**. (**A**) The protocol of gain-function of miR-185-5p and MI model was shown in Supplementary Figure S5A. (**B**) The protocol of loss-function of CatK and MI model was shown in Supplementary Figure S5C. Arterial densities in ischemic hearts were analyzed to obtain specific evidence of vascularity at the microcirculation level by performing double stainings of CD31 and α-SMA. Red, CD31. Green, α-SMA. Blue, DAPI. Scar bar is 50 μm. N = 10-15 per group. **P* < 0.05 *vs* Sham. ^#^*P* < 0.05 *vs* Negative agomir or scramble shRNA.

**Table S1. Demographic and clinical variables of control and ACS patients**

|  | Control | ACS | *P* value |
| --- | --- | --- | --- |
| Age (years) | 60±8 | 63±9 | 0.096 |
| Male, n (%) | 15(50) | 21(70) | 0.114 |
| BMI (kg/m^2^) | 23.4±3.9 | 25.6±4.8 | 0.152 |
| Gensini score | 19.6±10.7 | 104.5±37.6 | <0.001 |
| Hypertension, n (%) | 10(33.3) | 21(70) | 0.004 |
| Diabetes mellitus, n (%) | 4(13.3) | 11(36.7) | 0.037 |
| Smoking, n (%) | 11(36.7) | 18(60) | 0.071 |
| Previous myocardial infarction, n (%) | 4(13.3) | 0 |  |
| Previous angioplasty, n (%) | 0 | 5(16.7) |  |
| Previous bypass surgery, n (%) | 0 | 0 |  |
| Previous cerebrovascular disease, n (%) | 1(3.3) | 5 (16.7) | 0.085 |
| LDL (mmol/l) | 2.57±0.96 | 2.8±1.05 | 0.377 |
| HDL(mmol/l) | 1.30±0.33 | 1.02±0.25 | <0.001 |
| TG (mmol/l) | 1.83±1.32 | 1.82±0.61 | 0.968 |
| TC (mmol/l) | 4.41±1.23 | 4.47±1.45 | 0.859 |
| ARB/ACEI, n(%) | 11(36.7) | 23(76.7) | 0.002 |
| CCBs, n(%) | 8(26.7) | 15(50) | 0.063 |
| Mononitrate ester healer, n(%) | 10(33.3) | 16(53.3) | 0.118 |
| Beta blockers, n(%) | 18(60) | 27(90) | 0.07 |
| Statins, n(%) | 28(93.3) | 28(93.3) | 1 |

Data were expressed as mean ± standard deviation or the number (%) of patients. ACS, acute coronary syndrome. BMI, body mass index. LDL, low-density lipoprotein. HDL, high-density lipoprotein. TG, triglyceride; TC, total cholesterol. ARB, angiotensin receptor blocker. ACEI, angiotensin converting enzyme inhibitor. CCBs, calcium channel blockers.

**Table S2. Echocardiographic Parameters in animal experiments in mice treated with agomir**

|  | Sham | MI | MI + Negative agomir | MI + miR-185 agomir |
| --- | --- | --- | --- | --- |
| LVAWs (mm) | 1.15±0.15 | 0.66±0.12** | 0.69±0.1 | 0.35±0.05** |
| LVAWd (mm) | 0.69±0.04 | 0.49±0.07** | 0.51±0.08 | 0.31±0.03** |
| LVPWs (mm) | 1.25±0.18 | 0.9±0.1* | 0.99±0.21 | 0.6±0.06* |
| LVPWd (mm) | 0.8±0.1 | 0.65±0.05* | 0.67±0.08 | 0.49±0.02* |
| LVESD (mm) | 2.46±0.12 | 3.6±0.68* | 3.61±0.59 | 4.81±0.2* |
| LVEDD (mm) | 3.84±0.09 | 4.69±0.53* | 4.8±0.52 | 5.34±0.31 |
| LVESV ( μL) | 21.98±1.96 | 52.67±10.6* | 49±8.88 | 105.67±21.13** |
| LVEDV (μL) | 68.68±7.88 | 97.78±20.37* | 99.33±5 | 129.33±10* |
| LVSV (μL) | 45.33±3.06 | 35.4±1.44** | 36.93±1.0 | 19.8±1.7** |
| LVEF (%) | 67.5±6.5 | 46.31±5.48** | 49.78±7.5 | 18.55±10.4** |
| LVFS (%) | 37.4±5.42 | 23.28±3.16** | 25.29±4.46 | 8.39±5.03** |
| HR (bpm) | 488±7.5 | 505±5.5* | 503±2.5 | 506±2.0 |
| CO (ml/min) | 25±1.73 | 16.27±2.97** | 16.67±1.53 | 10.8±1.06** |

LVAWs indicates LV anterior wall, end systole; LVAWd, LV anterior wall, end diastole; LVPWs, LV posterior wall, end systole; LVPWd, LV posterior wall, end diastole; LVESD, LV end-systolic diameter; LVEDD, LV end-diastolic diameter; LVESV, LV end-systolic volume; LVEDV, LV end-diastolic volume; LVSV, LV stroke volume; LVEF, LV ejection fraction; LVFS, LV fractional shortening; HR, heart rate; CO, cardiac output

**Table S3. Echocardiographic Parameters in animal experiments in mice treated with adenovirus**

|  | Sham | MI | MI + Scramble shRNA | MI + CatK shRNA |
| --- | --- | --- | --- | --- |
| LVAWs (mm) | 1.17±0.08 | 0.98±0.11* | 0.96±0.07 | 0.36±0.07** |
| LVAWd (mm) | 0.74±0.05 | 0.59±0.04* | 0.8±0.12 | 0.33±0.07** |
| LVPWs (mm) | 1.3±0.08 | 1.0±0.11** | 0.98±0.08 | 0.69±0.11** |
| LVPWd (mm) | 0.89±0.04 | 0.57±0.04** | 0.65±0.09 | 0.51±0.07* |
| LVESD (mm) | 2.27±0.30 | 3.67±0.13** | 3.30±0.26 | 4.31±0.15** |
| LVEDD (mm) | 4.01±0.18 | 4.77±0.32* | 4.5±0.42 | 4.82±0.24 |
| LVESV ( μL) | 15.22±4.14 | 51.2±5.67* | 51.08±10.61 | 95.86±9.69** |
| LVEDV (μL) | 67.95±10.6 | 92.33±2.5* | 97.33±7.02 | 114±17.78 |
| LVSV (μL) | 48.67±1.53 | 32.97±1.0** | 34.17±1.61 | 21.67±1.53** |
| LVEF (%) | 78±2.65 | 43.67±4.16** | 44±3.6 | 23.67±3.51** |
| LVFS (%) | 43.33±4.73 | 22.87±2.8** | 26.67±3.51 | 10.57±1.91** |
| HR (bpm) | 479±9.0 | 507.5±2.5* | 503.6±3.5 | 507.5±4.0 |
| CO (ml/min) | 27.93±1.1 | 14.67±1.53** | 15±1.0 | 12.17±1.04** |

LVAWs indicates LV anterior wall, end systole; LVAWd, LV anterior wall, end diastole; LVPWs, LV posterior wall, end systole; LVPWd, LV posterior wall, end diastole; LVESD, LV end-systolic diameter; LVEDD, LV end-diastolic diameter; LVESV, LV end-systolic volume; LVEDV, LV end-diastolic volume; LVSV, LV stroke volume; LVEF, LV ejection fraction; LVFS, LV fractional shortening; HR, heart rate; CO, cardiac output.

**Table S4.** **Primer sets Used in the present study**

| **Gene names Forward (5’-3’) Reverse(5’-3’) Application** |
| --- |
| hsa-CatK ACACCCACTGGGAGCTATG GACAGGGGTACTTTGAGTCCA RT/PCR  hsa-Hes1 GGAGAGGCGGCTAAGGTGTT CGCTGTTGCTGGTGTAGACG RT/PCR  hsa-Hey1 AGAAGGAGAGTGCGGACGAG CGGCGCTTCTCAATTATTCC RT/PCR  hsa-Hey2 GAGCATAGGATTCCGAGAGTGC GCGCAAGTGCTGAGATGAGA RT/PCR  hsa-GAPDH CTGCACCACCAACTGCTTAG AGGTCCACCACTGACACGTT RT/PCR  mmu-CatK GGATACGTTACTCCAGTCAAGAACCAG GCCTCCACAGCCATAATTCTCAGTC RT/PCR  mmu-GAPDH AGGTCGGTGTGAACGGATTTG GAGTTGCTGTTGAAGTCGCA RT/PCR |
